# Dual inhibition of HERs and PD-1 counteract resistance in KRAS^G12C^-mutant head and neck cancer

**DOI:** 10.1186/s13046-024-03227-0

**Published:** 2024-11-20

**Authors:** Ofra Novoplansky, Sankar Jagadeeshan, Manu Prasad, Ksenia M. Yegodayev, Divyasree Marripati, Raghda Abu Shareb, Yariv Greenshpan, Sooraj Mathukkada, Talal Ben-Lulu, Baisali Bhattacharya, Angel Porgador, Dexin Kong, Johannes Brägelmann, J. Silvio Gutkind, Moshe Elkabets

**Affiliations:** 1 The Shraga Segal Department of Microbiology, Immunology and Genetics, Ben-Gurion University of the Negev, 84105 Beer-Sheva, Israel; 2 Faculty of Health Sciences, Ben-Gurion University of the Negev, Beer-Sheva, Israel; 3 School of Pharmaceutical Sciences, Tianjin Medical University, Tianjin, China; 4 Department of Translational Genomics, University of Cologne, Faculty of Medicine and University Hospital Cologne, 50937 Cologne, Germany; 5 Mildred Scheel School of Oncology, Faculty of Medicine and University Hospital Cologne, University of Cologne, 50937 Cologne, Germany; 6 University of Cologne, Faculty of Medicine and University Hospital Cologne, Center for Molecular Medicine Cologne, 50937 Cologne, Germany; 7 Department of Pharmacology and Moores Cancer Center, University of California San Diego, La Jolla, CA USA

**Keywords:** KRAS^G12C^ mutation, Adagrasib, Sotorasib, Drug resistance, Cell-autonomous, Tumor microenvironment, Head and neck cancer, HER signaling, PD-L1/PD1

## Abstract

**Background:**

Basket clinical trials targeting the KRAS^G12C^-mutation in solid tumors have shown initial promise, including in orphan KRAS^G12C^ head and neck cancer (HNC). However, development of resistance to KRAS^G12C^-mutant-specific inhibitors (KRAS^G12C^i) remains a major obstacle. Here, we investigated the intrinsic (tumor-cell autonomus) and tumor-microenvironment (TME) mechanisms of resistance to the KRAS^G12C^i—MRTX849 and AMG510 in a unique syngenic murine KRAS^G12C^-mutated HNC cell line.

**Methods:**

Western-blotting was used for protein abundance and activation, overexpression, and ligand activation studies to verify the intrinsic mechanism of resistance to KRAS^G12C^i in KRAS^G12C^-mutated HNC cell line, 4NQO-L. In vitro KRAS^G12C^-acquired-resistant cells were developed from 4NQO-L (4NQO-L-AcR). MRTX849/lapatinib combination efficacy, and CD8^+^ T-cells depletion, were assessed in C57BL/6 J mice and supplementation of anti-PD-1 (αPD-1) to MRTX849/lapatinib was also performed in 4NQO-L– KRAS^G12C^i-senisitve and 4NQO-L-AcR tumors. Immunohistochemistry (IHC) and Immunoflourescence (IF) analyses were performed to profile the TME and programmed death-ligand 1 (PD-L1) expression in tumors.

**Results:**

Activation and upregulation of EGFR and HER2/3 (pan-HERs) are the intrinsic mechanism of resistance to KRAS^G12C^i in 4NQO-L cells, and blocking pan-HERs signaling with lapatinib enhanced MRTX849 efficacy in vitro by inhibiting the MAPK and AKT/mTOR pathways. 4NQO-L-AcR upregulated the expression of pan-HERs, and lapatinib treatment re-sensitized 4NQO-L-AcR to MRTX849. In mice, MRTX849 showed a slight anti-tumor effect, but in combination with lapatinib a significant tumor growth delay was observed, but all tumors progressed over time. Histopathology analysis of the TME revealed infiltration of CD8^+^ T-cells after treatment combination, and these CD8^+^ T-cells play a key role in MRTX849/lapatinib efficacy. MRTX849/lapatinib treatment upregulated PD-L1 overexpression in both stromal and tumor cells, which presumably suppressed CD8^+^ T-cells and enabled immune escape and tumor progression. Supplementation of αPD-1 prolonged the progression-free survival of 4NQO-L-bearing mice treated with MRTX849/lapatinib. MRTX849/lapatinib treatment delayed tumor growth of 4NQO-L-AcR in mice; however, the percentages of CD8^+^ T-cells in 4NQO-L-AcR were low, and supplementation of MRTX849/lapatinib with αPD-1 did not improve the outcome.

**Conclusions:**

Our study highlights the critical need for blocking both intrinsic and extrinsic mechanisms of resistance for the prolonged response and shows that such treatment is ineffective in KRAS^G12C^i-AcR tumors.

**Supplementary Information:**

The online version contains supplementary material available at 10.1186/s13046-024-03227-0.

## Highlights of the study

1. Pan-HERs signaling mediates intrinsic resistance to KRAS^G12C^ inhibitors in head and neck cancer.
2. The efficacy of KRAS^G12C^ inhibitors is limited by the activation and upregulation of EGFR and HER2 in 4NQO-L cells.
3. Blocking pan-HERs signaling enhances MRTX849 efficacy in vitro by inhibiting the MAPK and AKT/mTOR pathways in both sensitive and KRAS^G12C^i-acquired-resistance 4NQO-L cells.
4. The MRTX849/lapatinib combination in mice results in delayed 4NQO-L tumor growth and CD8^+^ T-cell determine efficacy, and progression on this therapy combination is accompanied by PDL1 expression and accumulation of CAFs
5. Supplementation the MRTX849/lapatinib combination with αPD-1 prolongs the progression-free survival of tumor-bearing mice.
6. KRAS^G12C^i-acquired-resistant tumors are associated with an immune-desert and supplementation of αPD-1 does not improve therapy efficacy.

## Introduction

Head and neck cancer (HNC) is the seventh most prevalent cancer worldwide, and its incidence continues to rise, with a predicted 30% annual increase by 2030 [1]. It is widely recognized that management of HNC is challenging owing to the complexities involved in the treatment of the disease. In particular, conventional therapeutic approaches, including surgical intervention, radiation therapy, and chemotherapy, with its inherent cytotoxicity, have the potential to cause considerable disfigurement and impair the functionality of essential bodily structures. Moreover, mortality figures for advanced and metastatic HNC patients are alarmingly high, with over 50% of cases resulting in death within five years [2], representing a significant clinical challenge [3]. The poor prognosis and the lack of efficacy of current treatments thus highlight the need for new therapeutic approaches [4]

Basket trials in oncology expedite tumor-agnostic approvals, prioritizing promising treatments based on shared genetic mutations or biomarkers [5]. Such trails also allow treating patients. In HNC patients, the *HRAS* oncogene has garnered significant attention because of its frequent mutations, emphasizing its crucial function as a target molecule [6–12]. On the contrary *KRAS* mutations are infrequent in patients with HNC with ~ 0.5% in primary lesions [13, 14] and ~ 3.5% in advanced recurrent disease [13, 15]. These *KRAS*-mutated HNC patients are eligible for basket trials with KRAS^G12C^ inhibitors (KRAS^G12C^i) such as NCT05263986, NCT06166836, NCT04975256, NCT04380753, NCT03114319, NCT05162443, NCT04185883, NCT05119933, NCT06237400, NCT05178888, NCT06024174, NCT05002270, NCT06117371, NCT06006793, NCT06235983, NCT05367778, NCT06244771, NCT05768321, NCT04006301, NCT05462717, NCT05315180, and NCT04151342. Notably, the success in treating *KRAS*-mutated patients with the KRAS^G12C^i, such as AMG510 (sotorasib), MRTX849 (adagrasib), and RMC-9805 has ignited optimism among clinicians, despite that all patients develop therapy resistance over time.

Resistance to therapy is a complex process, with a range of intrinsic (tumor cell-autonomous) and extrinsic factors (related to the cells/factors from the tumor microenvironment (TME)) that limit therapy efficacy [9, 16]. Recent studies have shown that the development of drug resistance constitutes a significant obstacle to the treatment with KRAS^G12C^i, such as AMG510 and MRTX849 [17–27]. Intrinsic resistance to KRAS^G12C^i can stem from the activation of wild-type RAS by multiple receptor tyrosine kinases (RTKs) rather than just a single RTK [24, 28], while extrinsic mechanisms of resistance can be related to angiogenesis and coagulation pathways, as well as in fatty acid and xenobiotics metabolism along with reduced adaptive immune activity [21, 22]. While most studies investigated the either intrinsic or extrinsic mechanism of resistance to KRAS^G12C^i, we were interested in exploring the interplay between the intrinsic and extrinsic mechanism of resistance to KRAS^G12C^i in the only KRAS^G12C^ murine HNC model that is available.

In this study, we describe the response to KRAS^G12C^i in a murine HNC model and identify HER family activation as the key driver of intrinsic resistance. By studying the response to KRAS^G12C^i in an immune-competent model, we observed that the antitumor efficacy of dual treatment comprising an anti-HER therapy (lapatinib) and a KRAS^G12C^i (MRTX849 or AMG510) depends on CD8^+^ T cell activation, and that supplementation of the MRTX849/ lapatinib combination with anti-programmed cell death protein-1 (αPD-1) prolonged the progression-free survival of tumor-bearing mice. We further showed that KRAS^G12C^i –acquired resistant tumors induce immune-desert phenotype, and such tumors do not benefit from supplementation of αPD-1.

## Materials and methods

### Cell lines

The 4NQO-L (lip) and 4NQO-T (tongue) cell lines were developed in our laboratory [29] by exposing mice to drinking water containing 50 μg/mL 4-nitroquinoline 1-oxide (4NQO; N8141, Sigma, St. Louis, MO, USA) [29] All cell lines are maintained at 37 °C in a humidified atmosphere with 5% CO_2_ in Dulbecco's modified Eagle’s medium (DMEM) supplemented with 1% l-glutamine (200 mM), 100 units of penicillin and streptomycin, and 10% fetal bovine serum (FBS). Cells are routinely tested for mycoplasma infection and treated with appropriate antibiotics, as needed (De-Plasma™, TOKU-E, D022).

### Antibodies and reagents

Antibodies were purchased from the following suppliers: anti phospho p44/42 MAPK (pERK 1/2) (#4370S), anti p44/42 MAPK (ERK1/2) (#4695) anti phospho AKT Ser473 (#4060), anti phospho AKT Thr308 (#13,038), anti AKT (#4691), anti S6 (#2217L), anti phospho S6 S240/244 (#5364), anti-EGFR (#4267S), anti pEGFR (#2236L), anti β-actin (#4970L), anti-CD8 (#98941S), anti-HER2 (#2165S), anti-phospho HER2 Y1221/1222 (#2243S), anti-HER3 (#12708S), anti-phospho HER3 Tyr1289 (#4791S) from Cell Signaling Technology; anti Ki67 (#275R-15); Abcam: αSMA (ab5694), anti-PD-L1 (ab238697), anti-cytokeratin 14 (ab181595) from Cell Marque™; anti phospho S6 Ser235/236 (AF3918) from R&D systems; and anti-actin (08691001) from MP Biomedicals; αPD-1 (rat anti-mouse PD-1, BE0146-25 -clone RMP1-14), In vivo Plus™ anti-mouse CD8α (rat anti-mouse CD8α, BP0061-25 Clone 2.43), and IgG (rat anti-mouse IgG2a, BE0089-25) from Bio X Cell.

Inhibitors were purchased from following suppliers: AMG510 (HY-114277), MRTX849 (HY-130149), lapatinib (HY-50898), erlotinib (HY-50896), PHA665752 (HY-11107) afatinib (HY-10261) and Foretinib (HY-10338) from MedChemExpress; R428 (A13741-2) from AdooQ Bioscience. All inhibitors were dissolved in dimethyl sulfoxide (DMSO) for the in vitro studies.

### IC_50_ calculation

Cells were seeded in 96-well plates (3000–5000 cells per well), treated with increasing concentrations of the drug being tested, and allowed to proliferate for four days. At the endpoint, the cells were stained with crystal violet (1 g/L) for 10 min at room temperature. The crystal violet was then dissolved out in 10% acetic acid, and the absorbance was measured at 570 nm using a spectrophotometer (Epoch, Biotech). The IC_50_ values were calculated using GraphPad Software version 7.

### Western blotting

Cells were washed with cold phosphate-buffered saline (PBS) and scraped into lysis buffer supplemented with a phosphatase inhibitor cocktail (Stratech, B15001-BIT). Lysates were cleared by centrifugation at 14,000 rpm for 10 min at 4 °C, and the supernatants were collected and assayed for protein quantification using the Bradford protein assay (Bio-Rad, 5,000,006). The total lysate (20 μg) was resolved on NuPAGE 4–12% Bis–Tris gels and transferred electrophoretically to PVDF membranes (Bio-Rad, 1,704,159). Membranes were blocked for 1 h in 5% bovine serum albumin (BSA) in Tris-buffered saline (TBS)-Tween and then hybridized using the primary antibodies in 5% BSA TBS-Tween. Mouse and rabbit horseradish peroxidase-conjugated secondary antibodies (1:20,000, GE Healthcare) were diluted in 5% BSA in TBS-Tween. Protein-antibody complexes were detected by chemiluminescence with ECL (Westar Supernova, Cyanogen XLS3.0100, and Westar Nova 2.0 Cyanagen XLS071.0250), and images were captured using a c300 Azure camera system. Quantification was performed using Image J software. Protein level was calculated relative to loading control (actin) and presented as fold change vs. the control sample.

### Immunohistochemistry

Tumors were fixed in 4% paraformaldehyde solution overnight at room temperature and then maintained in 70% ethanol until they were embedded in paraffin. Paraffin-embedded tumor blocks were sectioned into 5-μm slices, loaded onto microscope slides, and deparaffinized at 60 °C for 1 h. After additional deparaffinization with a xylene substitute (Leica, 3803672E) and rehydration in a descending alcohol series, antigen retrieval was performed. The slides were incubated in 10 mM citric acid buffer (pH 6.0) at 100 °C for 15 min, cooled in buffer at room temperature, and rinsed with doubly distilled water for 3 min, × 3. Thereafter, endogenous peroxidases were inactivated using 0.3% hydrogen peroxide in methanol buffer for 30 min. The slides were washed three times with PBS for 3 min and then blocked with 5% BSA in PBS-T (0.1% Tween) for 1 h at room temperature. Slides were then incubated overnight at 4 °C with the primary antibodies, Ki67 (Sigma, AB9260, 1:200), CD8 (Cell Signaling #98941S 1:200), αSMA (AbCam, ab5694, 1:100), and anti PD-L1 (AbCam, ab238697, 1:100), diluted in blocking solution. The following day, the slides were washed three times with PBS-T. An ABC kit (VECTASTAIN®ABC, VE-PK-6200) was used for detection according to the manufacturer's protocol, with 3,3'-diaminobenzidine (DAB) (ACH500-IFU; ScyTek Laboratories) as a substrate for color development. The slides were counterstained with hematoxylin, dehydrated, and mounted in mounting medium (Sub-X, Leica 3,801,740). All slides were digitalized using a Panoramic Scanner (3DHISTECH, Budapest, Hungary), and the analysis was performed using Qupath-0.2.3 software. The fields for tumor tissue analysis were chosen by a blinded investigator. Cells within the analysis field were detected using Qupath-0.2.3 software and were defined as positive or negative for DAB staining according to a threshold set by two independent blinded investigators. Cell detection criteria and thresholds were maintained between the slides. The specificity of the staining and analysis threshold was verified by comparison with a matched negative control tissue, which was incubated without a primary antibody but was subjected to all secondary antibody development processes.

### Immunofluorescence

The tissue was processed as described above and then incubated with the primary antibodies PD-L1 (ab238697, 1:100) and cytokeratin-14 (AB-ab181595-100 1:2000) overnight at 4 °C. The next day, cells were rinsed with PBS-T and incubated with Alexa Fluor-647 Anti-rabbit secondary antibody (Jackson ImmunoResearch, PA, USA, 111–605-144, 1:250) or Alexa Flior-488 Anti-mouse secondary antibody (Jackson ImmunoResearch, 115–545-062, 1:250) at room temperature for 1 h. Finally, the cells were washed with PBS-T and mounted with DAPI Fluoromount-G® (SouthernBiotech, Birmingham, CA, USA, 0100–20).

### Cell proliferation assay

Cells were seeded in a 24-well plate (10,000–20,000 cells per well) and treated the following day. At the end of the experiment, cells were stained with crystal violet (1 g/L). Quantification was performed by dissolving out the crystal violet (10% acetic acid) and reading the optical density at 570 nm using a spectrophotometer (Epoch, Biotech).

### In vivo experiments

Mice for the in vivo experiments were maintained and treated in accordance with the institutional guidelines of the Ben-Gurion University of Negev. Animal experiments were approved by the Institutional Animal Care and Use Committee (IL-37–10-2022E). Mice were housed in air-filtered laminar flow cabinets with a 12-h light/dark cycle and food and water ad libitum. At the end of the experiment, the animals were euthanized with CO_2_. To generate tumor-bearing mice, cells (5 × 10^6^) were suspended in 100 μl of PBS and injected s.c. into the right and left flanks of 6-week-old male C57BL/6 J (WT) mice (Envigo, Huntingdon, UK, C57/BL/6). For the orthotopic model, cells (5 × 10^6^) were suspended in 50 μl of PBS and injected into the lips of 6-week-old male C57BL/6 J (WT) mice (Envigo, Huntingdon, UK, C57/BL/6) or 6-week-old male NSG mice (NOD.Cg-Prkdcscid Il2rgtm1Wjl/SzJ, Jackson Labs, Bar Harbor, ME, USA). Tumor-bearing mice were then randomized into groups based on the tumor volume (between 150 and 200 mm^3^ for tumors in the flanks and 30–50 mm^3^ for orthotopic tumors in the lips). For all *in-vivo* experiments, MRTX849 (30 mg/kg) [30] and lapatinib (50 mg/kg) [31, 32] were dissolved in 5% DMSO, 5% Tween 80, 40% PEG 300, and 50% PBS. The drugs were administered daily by oral gavage. Vehicle-treated mice received 5% DMSO, 5% Tween 80, 40% PEG 300, and 50% PBS. For efficacy experiments with MRTX849/lapatinib and αPD-1 antibodies, MRTX849 (30 mg/kg/day)/lapatinib (50 mg/kg/day), αPD-1 and IgG were used at a concentration of 100 µg/mouse. Tumors were measured twice a week using a digital caliper, and the tumor volume was calculated according to the formula V = (L × W × W) π/2, where V is the tumor volume, W is the tumor width, and L is the tumor length. Tumor volumes are plotted as means ± SEM.

### CD8^+^ depletion experiments

In vivo Plus™ anti-mouse CD8α or IgG (In vivo Plus™ rat IgG2b) isotype controls, both from Bio X Cell, were used for CD8 depletion experiments. The animals were injected intraperitoneally with 1 mg/mouse of αCD8 antibody or IgG two days before tumor cell injection. When the tumors reached ~ 70 mm^3^, the mice were randomized into treatment groups. MRTX849 (30 mg/kg) with lapatinib (50 mg/kg treatment was continued with 500 µg/mouse of αCD8 antibody or IgG every 5 days.

### Statistical analysis

Statistical analysis was performed using GraphPad Prism software versions 7 and 9, and the results are presented as means ± SEM. For comparisons between two groups, *P* values were calculated using the unpaired t-test (*p < 0.05, **p < 0.01, ***p < 0.001). For comparisons between three or more groups, *P*-values were calculated using one-way ANOVA.

## Results

### KRAS^G12C^i treatment hyperactivates HER signaling in tumor cells that attenuate the efficacy of MRTX849 and AMG510 in a KRAS^G12C^-HNC model

**Fig. 1:**
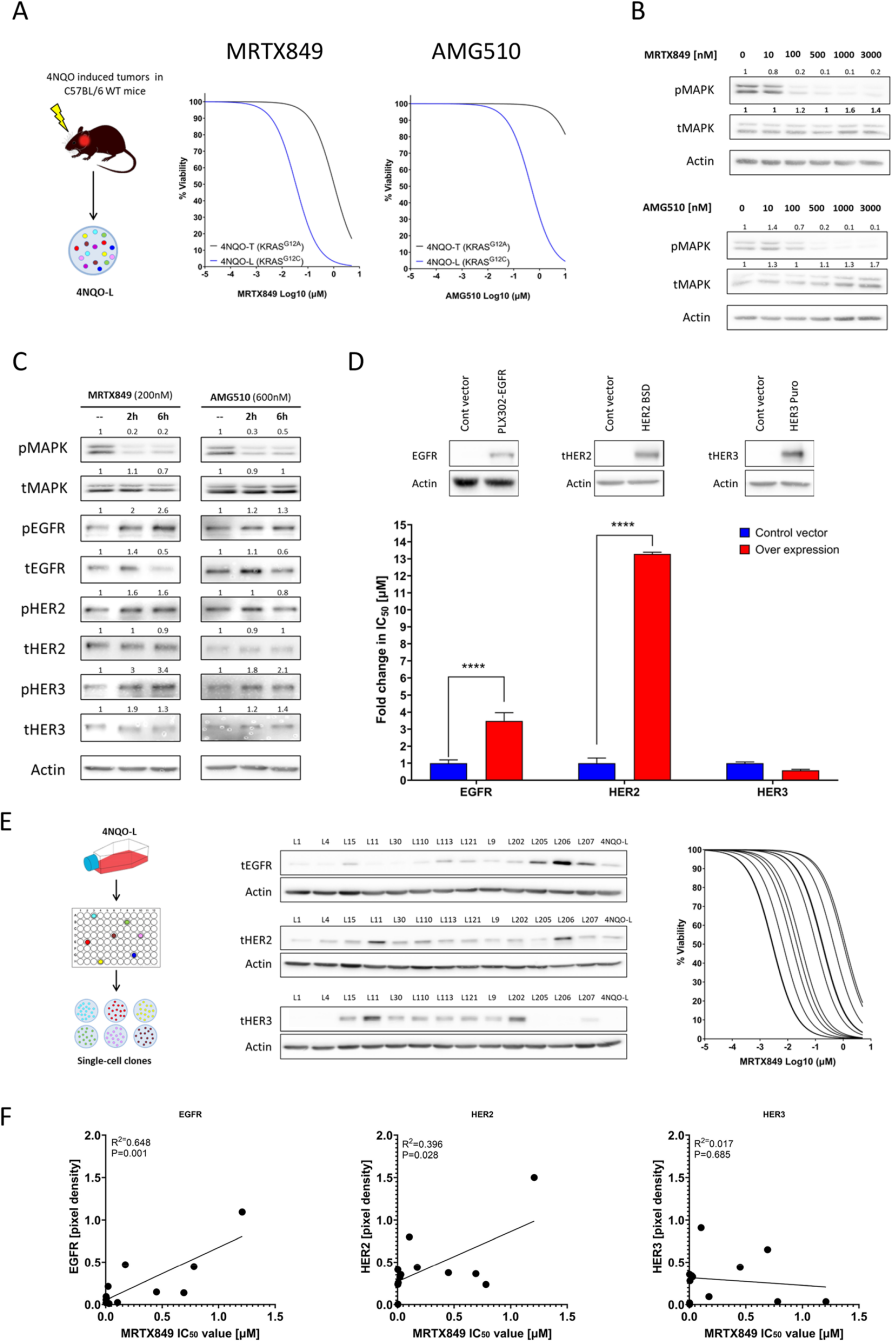
KRAS^G12C^i hyperactivates HER signaling and impairs efficacy of MRTX849 or AMG510 in KRAS^G12C^-HNC. **A** Left: Scheme showing the generation of the 4NQO-L cell line (or similarly 4NQO-T). Right: Cell viability graph indicating the sensitivity of the 4NQO-L cell line to MRTX849 or AMG510 after 96 h of treatment. Data are taken from three representative independent experiments. **B** Western blot of the indicated proteins following treatment of 4NQO-L cells with various concentrations of MRTX849 or AMG510. Numbers indicate the fold-change in protein levels normalized to actin. Data are taken from three representative independent experiments. **C** Western blot analysis of the indicated proteins at 2 and 6 h after treatment of 4NQO-L cells with MRTX849 (200 nM) or AMG510 (600 nM). Numbers indicate the fold-change in protein levels normalized to actin. Data are taken from three representative independent experiments. **D** Western blotting confirming the overexpression of EGFR, HER2, and HER3 in the 4NQO-L cell line and the corresponding fold changes in IC_50_ values indicating resistance to MRTX849 at 96 h following treatment. Data are taken from three representative independent experiments. Statistical significance was calculated using unpaired t-tests (****p < 0.0001). **E** Left: Scheme showing the generation of single-cell clones. Middle: Western blot quantification of the expression levels of EGFR, HER2, and HER3 in these single-cell clones. and Right viability graphs of these single-cell clones, indicating sensitivity to MRTX849. **F** Graph showing correlation between EGFR, HER2, and HER3 with the MRTX849 IC_50_ values of single-cell clones

To investigate the sensitivity of KRAS^G12C^i in HNC cells, we utilized two well-defined HNC cell lines developed in our laboratory: 4NQO-Lip (4NQO-L) and 4NQO-Tongue (4NQO-T) [29]. Genomic analyses revealed that the 4NQO-L and 4NQO-T cell lines harbor mutations in the KRAS genes, KRAS^G12C^ and KRAS^G12A^, respectively [29]. Comparing the response of the two cell lines to the KRAS^G12C^i, MRTX849 (adagrasib) and AMG510 (sotorasib), showed that the 4NQO-L cell line is significantly more sensitive than the 4NQO-T cell line to the two KRAS^G12C^i (Fig. 1A). The IC_50_ values for MRTX849 and AMG510 for the KRAS^G12C^ mutant 4NQO-L were 0.03374 and 0.4662 μM, respectively, and those for the KRAS^G12A^ mutant 4NQO-T were 1.025 and 43.57 μM, respectively (Supplementary Table 1). As expected, treatment with either MRTX849 or AMG510 resulted in a dose-dependent inhibition of the MAPK pathway, as indicated by pMAPK levels in the 4NQO-L cell line but not in the KRAS^G12A^ -4NQO-T cell line (Fig. 1B and Figure S1A).

Since the acquisition of resistance to KRAS^G12C^i is known to be mediated by upregulation and activation of the human epidermal growth factor receptor (HER) family members [17, 23–25, 33, 34] we investigated their role in the efficacy of KRAS^G12C^i in our KRAS^G12C^-4NQO-L cell line. Specifically, we examined the expression and activation status of the three major HER receptors, HER1/EGFR, HER2, and HER3, following treatment of the cells with MRTX849 or AMG510. Western blotting showed rapid activation (within 2 h) of all three receptors following MRTX849 treatment (less so, for AMG510) (Fig. 1C). To test whether overexpression of these receptors would limit the efficacy of KRAS^G12C^i, we overexpressed each HER family member in the KRAS^G12C^i-sensitive 4NQO-L cell line and determined the respective IC_50_ values for MRTX849. The IC_50_ values for EGFR-, HER2 and HER3 overexpressing 4NQO-L cells were 3.343, 1.777, and 0.00079 μM, respectively (Supplementary Table 1). The fold change in the IC_50_ values in the HER-overexpressing 4NQO-L cells (Fig. 1D) indicated that overexpression of EGFR or HER2, but not HER3, limited the efficacy of MRTX849.

To further explore the association between the baseline expression of EGFR, HER2, and HER3 and sensitivity to KRAS^G12C^i, we isolated single-cell clones from 4NQO-L cells by using limiting dilution seeding in 96-well plates (Fig. 1E, left), and then analyzed the expression of the three receptors by western blotting (Fig. 1E, middle) and determined their IC_50_ values for MRTX849 (Fig. 1E, right and Supplementary Table 1). A significant correlation was observed between sensitivity to MRTX849 (IC_50_ value) and endogenous expression of EGFR (*R* ^2^ = 0.648, *p* = 0.001) and HER2 (*R* ^2^ = 0.396, *p* = 0.028) (Fig. 1F). HER3 expression levels did not correlate with sensitivity to MRTX849 (*R* ^2^ = 0.017, *p* = 0.685) (Fig. 1F). Notably, the clones showed similar trends in their responses to AMG510 and MRTX849 (Figure S1B).

### Inhibition of EGFR/HER2 by lapatinib improves MRTX849 efficacy in vitro by blocking MAPK and AKT/mTOR signaling

**Fig. 2:**
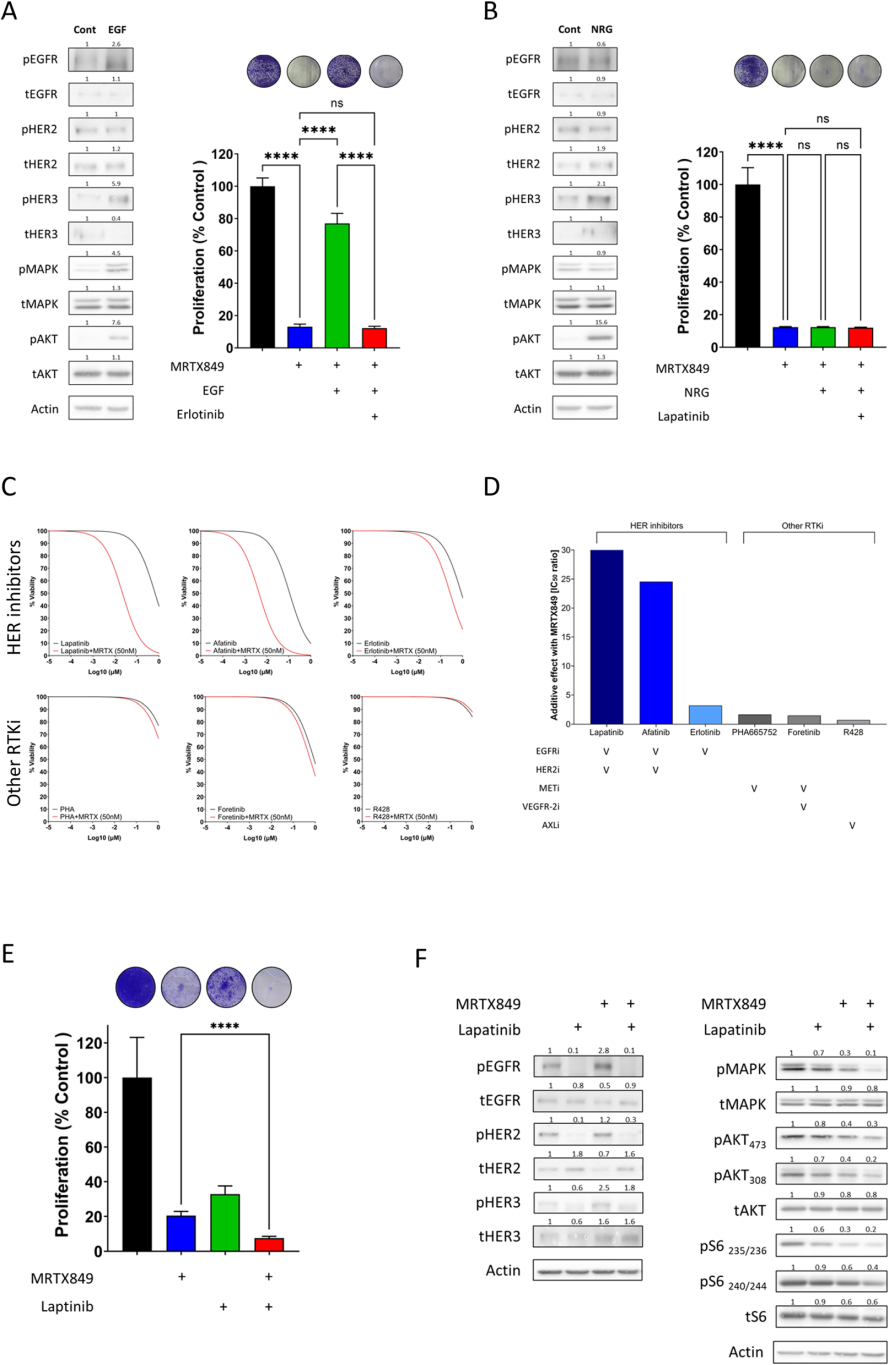
Ligand activation of RTKs limits MRTX849 efficacy, whereas pan-HER inhibition improves efficacy in vitro by blocking MAPK and AKT/mTOR signaling. **A** Left: Western blot analysis of the indicated proteins following stimulation of 4NQO-L cells with epidermal growth factor (EGF). Data are taken from two representative independent experiments. Right: Representative images showing crystal violet-stained plates with or without EGF stimulation during treatment with MRTX849 or erlotinib. Graph shows the proliferation rate of cells under the various treatment conditions normalized to control. Error bars indicate SD. Statistical significance was calculated using one-way ANOVA (****p < 0.0001), ns denotes not significant. **B** Left: Western blot analysis of the indicated proteins following NRG stimulation of 4NQO-L cells. Data are taken from two representative independent experiments. Right: Representative images showing crystal violet-stained plates with or without NRG stimulation during treatment with MRTX849 or lapatinib. Graph shows the proliferation rate of cells under various treatment conditions normalized to control. Error bars indicate SD. Statistical significance was calculated using one-way ANOVA (****p < 0.0001), ns denotes not significant. **C** Viability graphs indicating the sensitivity to MRTX849 in combination with various RTK inhibitors after 96 h of treatment in 4NQO-L cells. **D** Graphs showing the additive effects in 4NQO-L cells of various RTK inhibitors in combination with MRTX849. **E** Graph showing the proliferation rate of 4NQO-L cells in the presence of MRTX849, lapatinib, and MRTX849/lapatinib. Representative images of crystal violet-stained plates under various treatment conditions are shown. Error bars indicate SD. Error bars indicate SD. Statistical significance was calculated using unpaired t-tests (****p < 0.0001). **F** Western blot confirming the inhibition of pan-HER activation and subsequent blocking of the MAPK/AKT/mTOR pathway during MRTX849/lapatinib combination treatment of 4NQO-L cells. Data are taken from three representative independent experiments. Numbers indicate the fold-change in protein levels normalized to actin. Statistical significance was calculated using one-way ANOVA (****p < 0.0001) and unpaired t-tests (****p < 0.0001)

To assess whether activation of HER receptors contributes to resistance against KRAS^G12C^i, we treated 4NQO-L cells with MRTX849 in the presence or absence of recombinant epidermal growth factor (EGF) or neuregulin-beta 1 (NRG) to activate EGFR and HER3, respectively (Figs. 2A and B). EGF stimulation led to the activation of EGFR and the subsequent activation of the AKT and MAPK pathways (Fig. 2A left), thereby limiting the efficacy of MRTX849; the inactivation of AKT and MAPK pathway by EGF was achieved by supplementation of the culture with the EGFR inhibitor erlotinib (Fig. 2A right). Conversely, stimulation with the HER3 ligand NRG, which activates HER3, primarily stimulated the AKT pathway (Fig. 2B, left), but not MAPK, and failed to rescue 4NQO-L cells from MRTX849 (Fig. 2B right).

To further define the contribution of HER activation in limiting the efficacy of KRAS^G12C^i in the 4NQO-L model, we evaluated the ability of HER inhibitors – in comparison with other receptor tyrosine kinase inhibitors (RTKi) – to enhance MRTX849 efficacy. To this end, we investigated the effect on 4NQO-L of: erlotinib (a reversible inhibitor of both wild-type and common EGFR mutations) [35–37] lapatinib (an inhibitor of EGFR and HER2/3) [38, 39] afatinib (an irreversible inhibitor of wild-type and mutant EGFR, HER2, and HER4) [40] PHA665752 (an inhibitor of c-MET) [41] R428 (an inhibitor of AXL) [42] and foretinib (an inhibitor of MET, RON, AXL, and vascular endothelial growth factor receptors) [43] Specifically, we treated 4NQO-L cells with different concentrations of HER inhibitors and other RTKi, in the presence and absence of 50 nM MRTX849, and calculated the IC_50_ of each drug (Fig. 2C). The IC_50_ values for erlotinib, lapatinib, afatinib, PHA665752, R428 and foretinib (in absence of MRTX849) for 4NQO-L cells are 0.87 μM, 0.66 μM, 0.10 μM, 3.39 μM, 5.34 μM, and 0.88 μM. In the presence of 50 nM MRTX849, the IC_50_ values are 0.27 μM (+ erlotinib), 0.021 μM (+ lapatinib), 0.004 μM (+ afatinib), 2.0 μM (+ PHA665752), 7.39 (+ R428) and 0.58 μM (+ foretinib) (Supplementary Table 1). The ratio between the IC_50_ of the RTKi with and without MRTX849 is presented in Fig. 2D. These drug studies demonstrated that the sensitization of 4NQO-L tumor cells to MRTX849 by HER inhibitors (lapatinib, afatinib, and erlotinib) was superior to that of MET or AXL inhibitors, and thus further support the findings that EGFR and HER2 play a role in limiting the efficacy of KRAS^G12C^i. Moreover, these results suggest that, in combination with MRTX849, targeting both the EGFR and HER2/3 signaling pathways has greater anti-tumor activity compared to targeting EGFR (with erlotinib) alone. Because lapatinib was the most potent drug sensitizing MRTX849 cells, we decided to continue this line of research by validating the effect of the MRTX849/lapatinib combination in vitro and in mice (described in different sections below).

The experimental design for the in vitro validation of the effect of MRTX849/lapatinib treatment comprised a crystal violet proliferation assay (Fig. 2E), followed by profiling of the signaling pathways in 4NQO-L cells by western blot analysis (Fig. 2F). To this end, cell lysates were analyzed 6 h after treatment of the cells with MRTX849, lapatinib, or a combination of the two drugs. As expected, lapatinib prevented MRTX849-induced hyperphosphorylation of EGFR, HER2, and HER3 in 4NQO-L cells (Fig. 2F). Moreover, while monotherapy with MRTX849 primarily inhibited MAPK, supplementation with lapatinib inhibited the AKT and mTOR pathways alongside inhibition of the MAPK pathway (Fig. 2F). These results were confirmed with AMG510 in place of MRTX849 (Figure S2). Taken together, these results suggest that the activation of HER2 and EGFR limit the efficacy of MRTX849 or AMG510 and that the inhibition of these receptors is critical for prolonged anti-proliferative effects.

### Lapatinib sensitizes HER-overexpressed KRAS^G12C^i -acquired resistance 4NQO-L tumor cells

**Fig. 3:**
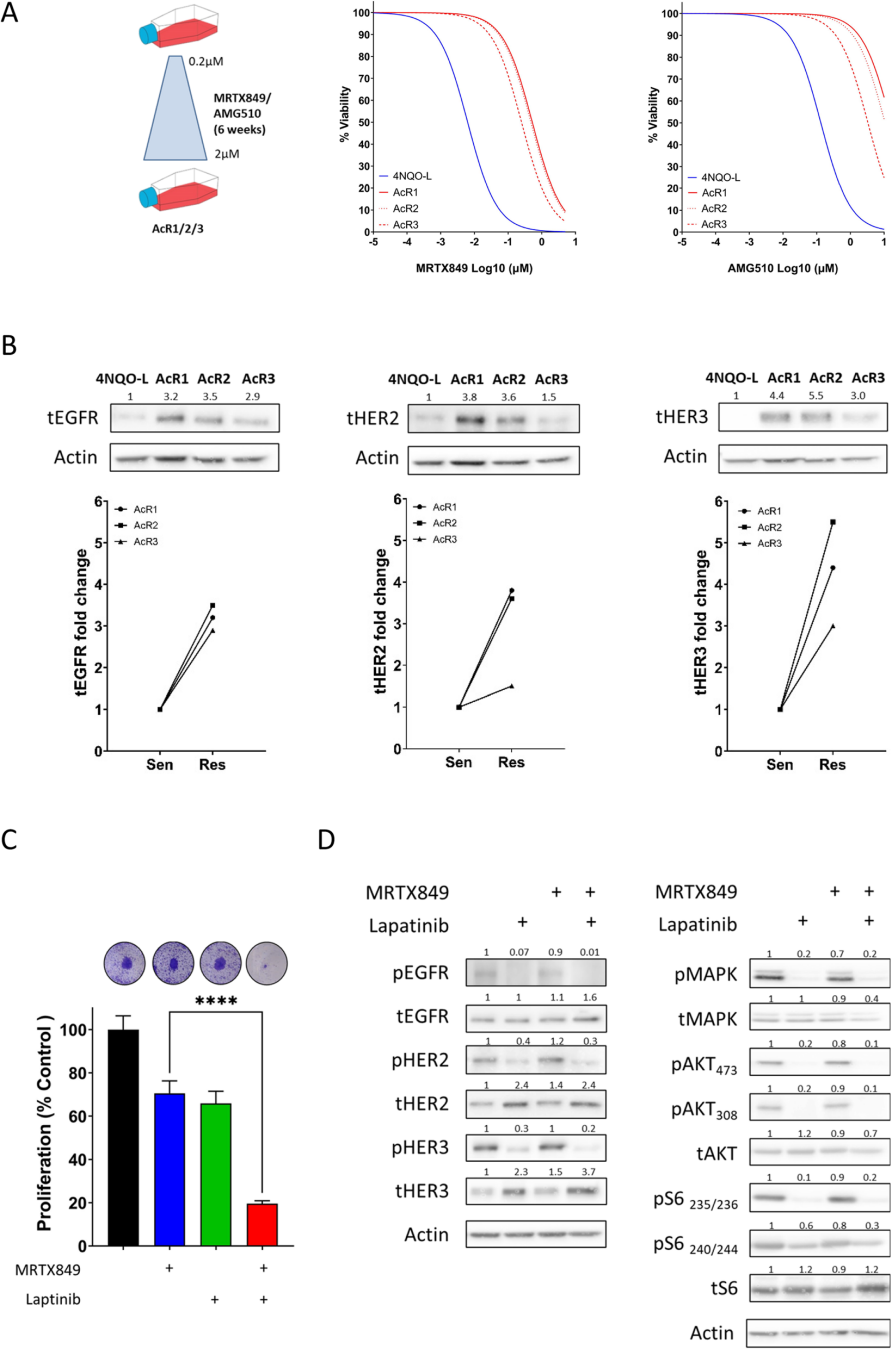
Lapatinib sensitizes HER-overexpressed KRAS^G12C^i -acquired resistant 4NQO-L tumor cells to MRTX849 treatment. **A** Left: Scheme showing the generation of MRTX849 or AMG510 resistant cell lines after treating the cells with increasing doses of the drug for 6 weeks. Right: Graph showing the viability of resistant cell lines after MRTX849 treatment. **B** Western blotting confirming the overexpression of EGFR, HER2 and HER3 in KRAS^G12C^i-resistant lines. Data taken from three representative independent experiments. Graph showing the fold-change in HER expression in KRAS^G12C^i-resistant lines. Numbers indicate the fold-change in protein levels normalized to actin. **C** Proliferation graph of a KRAS^G12C^i-resistant line in response to MRTX849, lapatinib, and MRTX849/lapatinib treatment. Error bars indicate SD. Statistical significance was calculated using unpaired t-tests (****p < 0.0001). **D** Western blot confirming the inhibition of pan-HER activation and subsequent blocking of the MAPK and AKT/mTOR pathway during MRTX849/lapatinib combination treatment in 4NQO-L acquired-resistance cells. Data are taken from three representative independent experiments

To explore whether the overexpression of HER family members is involved in the acquisition of resistance to KRAS^G12C^i, we generated resistant cell lines by exposing 4NQO-L cells to MRTX849 or AMG510 for 6 weeks until the tumor cells recovered their proliferative capability (Fig. 3A left). The resultant MRTX849-resistant cell line was designated 4NQO-L-AcR1 and the AMG510-resistant cell lines were designated 4NQO-L-AcR2 and 4NQO-L-AcR3. The IC_50_ for MRTX849 and AMG510 was increased in the three acquired resistance models, 4NQO-L-AcR1, -AcR2, and -AcR3, being 0.5225, 0.4755 and 0.2504 µM for MRTX849, respectively, and 16.08, 10.69, and 3.32 μM for AMG510, respectively (Fig. 3A right) (Supplementary Table 1). In addition, the cells showed cross-resistance between MRX849 and AMG510. Western blot analysis of the 4NQO-L-AcR acquired-resistance clones demonstrated upregulation of EGFR, HER2, and HER3 compared to the 4NQO-L parental line, as expected (Fig. 3B). In accordance with the IC_50_ value and western blot data, the 4NQO-L-AcR3 acquired-resistance clone exhibited the least resistance to MRTX849 as well as AMG510, as shown by the mild upregulation of the HER receptors; 4NQO-L-AcR1 and -AcR2 exhibited higher resistance, with more than a threefold increase in the expression of all three HERs analyzed. To explore the impact of MRTX849/lapatinib treatment on acquired-resistance cell lines, we first confirmed the superior antitumor activity of MRTX849/lapatinib by using a crystal violet proliferation assay (Fig. 3C), followed by profiling of the signaling pathways in 4NQO-L-AcR1 cells by western blot analysis (Fig. 3D). Cell lysates were examined 6 h after treatment with MRTX849, lapatinib, or a combination of the two drugs. As anticipated, lapatinib treatment prevented MRTX849-induced hyperphosphorylation of EGFR, HER2, and HER3 in 4NQO-L-AcR1 cells (Fig. 3D). Exposure of the cells to lapatinib resulted in powerful suppression of the MAPK, AKT, and mTOR signaling pathways in a manner analogous to the effects observed in the responsive 4NQO-L cells (Fig. 2F). Taken together, these results provide further support for the conclusion presented above that activation of HER2 and EGFR impairs the efficacy of MRTX849, and that inhibition of the receptors is essential for sustaining long-lasting antiproliferative effects.

### The efficacy of the MRTX849/lapatinib combination is dependent on CD8^+^ T-cells

**Fig. 4:**
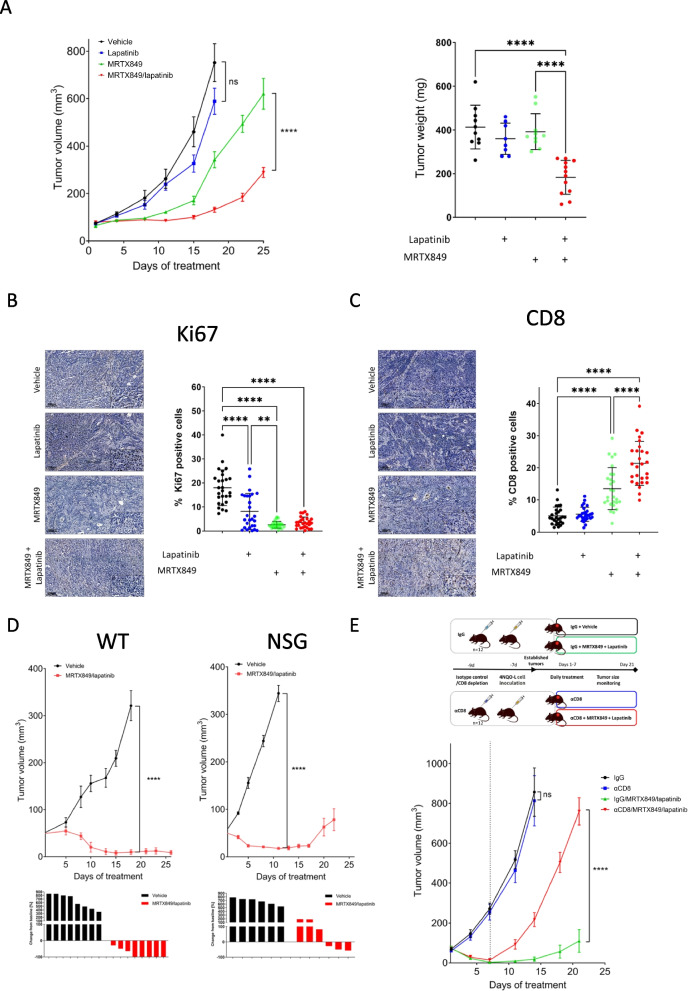
Additive effect of the MRTX849/lapatinib combination on 4NQO-L sensitive tumors is modulated by the presence of CD8^+^ T-cells. **A** Growth curve (left) and tumor weight (right) of 4NQO-L tumors in WT mice treated with vehicle (*n* = 5 mice, 10 tumors), MRTX849 (*n* = 5 mice, 10 tumors), lapatinib (*n* = 5 mice, 10 tumors), or the MRTX849/lapatinib combination (*n* = 6 mice, 12 tumors). Error bars indicate SEM. Statistical significance was calculated using one-way ANOVA (****p < 0.0001), ns denotes not significant. **B** IHC images showing the expression of Ki67 in tissue sections of 4NQO-L tumors treated with vehicle MRTX849, lapatinib, or the MRTX849/lapatinib combination (scale bars: 100 µm; inset 10 µm). *n* = 4 tumors and *n* = 27 analysis fields. Error bars indicate SD. Statistical significance was calculated using one-way ANOVA (**p < 0.01, ****p < 0.0001). **C** IHC images showing the infiltration of CD8^+^ T-cells in the tissue sections of 4NQO-L tumors treated with vehicle, MRTX849, lapatinib or the MRTX849/lapatinib combination (scale bar: 100 µm; inset: 10 µm). *n* = 4 tumors and *n* = 27 analysis fields. Error bars indicate SD. Statistical significance was calculated using one-way ANOVA (****p < 0.0001). **D** Top: Tumor volume of the orthotopic 4NQO-L tumors in WT mice (left; *n* = 8 mice, 8 tumors) or NSG mice (right; *n* = 6 mice, 6 tumors) treated with vehicle or the MRTX849/lapatinib combination. Statistical significance was calculated using one-way ANOVA (****p < 0.0001) Error bars indicate SEM. Bottom: Change in tumor volume from first to last day of treatment. **E** Top: Scheme of the experiment investigating the effect of CD8^+^ T-cell depletion on MRTX849/lapatinib efficacy. Bottom: Growth of 4NQO-L tumors in WT mice treated with IgG (*n* = 6 mice, 11 tumors), αCD8 T cells depletion (*n* = 6 mice, 12 tumors), IgG/MRTX849/lapatinib (*n* = 5 mice, 10 tumors) or αCD8/MRTX849/lapatinib (*n* = 6 mice, 12 tumors). Statistical significance was calculated using one-way ANOVA (****p < 0.0001), ns denotes not significant

To evaluate the antitumor activity of MRTX849, lapatinib, and their combination in vivo, 4NQO-L cells were implanted subcutaneously (*s.c*.) into C57BL/6 mice. When tumors reached ~ 70 mm^3^, tumor-bearing mice were randomized into four groups and treated daily with vehicle, MRTX849 (30 mg/kg/day), lapatinib (50 mg/kg/day), or a combination of the two. Monitoring of tumor growth kinetics for 25 days showed that MRTX849 monotherapy induced a modest delay in tumor progression, with a stable disease for about 7 days, while immediate progression was observed in tumor-bearing mice treated with lapatinib monotherapy. Notably, tumor-bearing mice treated with MRTX849/lapatinib exhibited stable disease for 14 days, but all tumors eventually progressed to the therapeutic combination. Analysis of tumor volume after 25 days of treatment showed that MRTX849/lapatinib treatment was three times more potent in delaying tumor progression (average tumor volume of 600 mm^3^ for MRTX849 compared to 200 mm^3^ for MRTX849/lapatinib treatment) (Fig. 4A left). This delay in tumor progression was also reflected in the tumor mass (Fig. 4A right). In addition, immunohistochemical (IHC) analysis of the tumors after three days of treatment showed reduced tumor cell proliferation in mice treated with MRTX849/lapatinib, as indicated by Ki67 staining (Fig. 4B).

Due to accumulating evidence that response to KRAS^G12C^i is modulated by the presence of CD8^+^ T cells in the TME [44–46] we utilized our syngeneic immune-competent mouse model to analyze the effects of treatment on the accumulation of cytotoxic lymphocytes (CD8^+^ T-cells) before and 3 days after treatment with MRTX849, lapatinib, and a combination of the two. Staining of tumors with anti-CD8 showed that MRTX849 treatment induced CD8^+^ T-cell infiltration and the combination with lapatinib further enhanced the infiltration (Fig. 4C). To assess the role of the immune system in the response to MRTX849/lapatinib, we injected 4NQO-L cells into the lip (orthotopically) of immunocompromised NSG and immunocompetent WT mice. We then compared tumor growth between mice treated with MRTX849/lapatinib and those receiving a vehicle control. In the NSG mice, all tumors initially responded to the combination treatment; however, 50% of the tumors progressed and doubled in size within 25 days. In contrast, in WT mice, all tumors showed a significant response to the treatment, shrinking in size compared to their baseline measurements (Fig. 4D). These results motivated us to further explore the impact of CD8^+^ T-cells on the antitumor effect of the MRTX849/lapatinib combination treatment by conducting a CD8^+^ T-cell depletion experiment in mice. To this end, we administered an anti-CD8 (αCD8) depleting antibody, or isotype control (IgG), to mice two days before the tumor implantation, and then confirmed the depletion efficiency by flow cytometry (Figure S3). When tumors reached ~ 70 mm^3^ each group (IgG or αCD8) was divided into two arms, with one arm receiving vehicle and the other receiving MRTX849/lapatinib combination treatment for seven days to allow treatment-induced infiltration (Fig. 4E). Monitoring of the tumor size revealed that CD8^+^ T-cell depletion alone did not exert any significant effect on tumor growth compared to the IgG control. However, in the groups treated with the MRTX849/lapatinib combination, the tumors in the mice that had undergone CD8^+^ depletion grew significantly faster than those in mice with intact CD8^+^ T-cells (Fig. 4E). Taken together, these results indicate that lapatinib enhances the efficacy of MRTX849 by reducing tumor cell proliferation and enhancing the infiltration and antitumor activity of CD8^+^ T-cells.

### Anti-PD-1 treatment enhances the efficacy of the MRTX849/lapatinib combination in mice

**Fig. 5:**
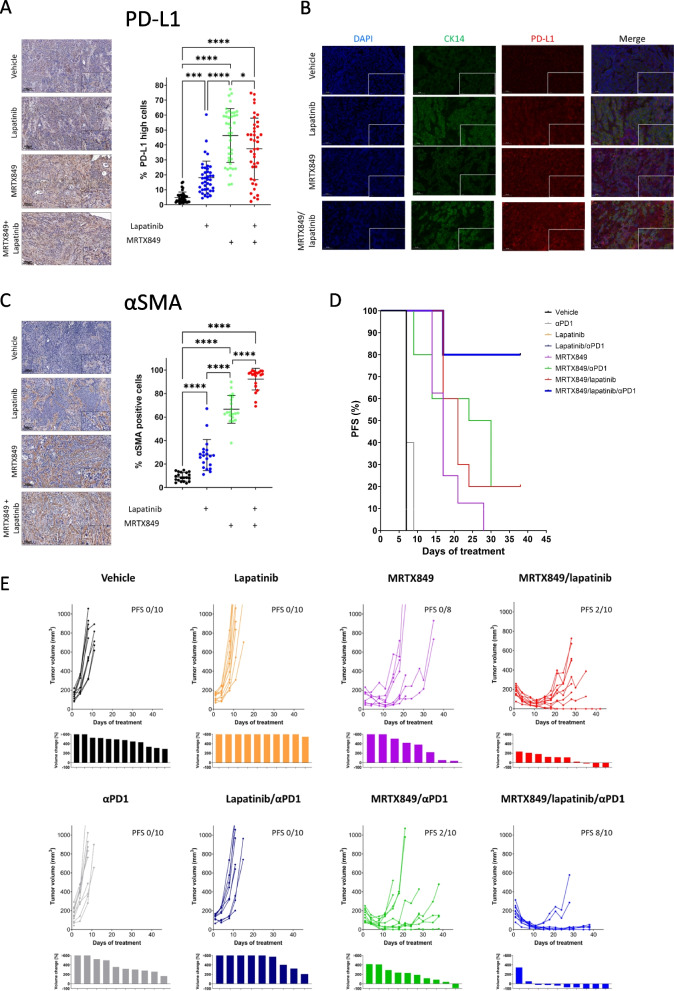
MRTX849/lapatinib/αPD1 treatment leads to prolonged tumor growth arrest in MRTX849-responsive tumors. **A** IHC images showing the expression of PD-L1 in the tissue sections of 4NQO-L tumors treated with vehicle, MRTX849, lapatinib, or the MRTX849/lapatinib combination (scale bars: 100 µm; inset 10 µm). *n* = 4 tumors and *n* = 40 analysis fields. Error bars indicate SD. Statistical significance was calculated using one-way ANOVA (*p < 0.05, ***p < 0.001, ****p < 0.0001). **B** Immunofluorescence staining of nuclei (DAPI), cytokeratin-14 (green), and PD-L1 (red) and merged images of the tissue sections of 4NQO-L tumors treated with vehicle, MRTX849, lapatinib, or MRTX849/lapatinib (scale bars: 50 µm; inset 10 µm). **C** IHC images showing the expression of αSMA in tissue sections of 4NQO-L tumors treated with vehicle, MRTX849, lapatinib, or MRTX849/lapatinib (scale bars: 100 µm; inset 10 µm). *n* = 4 tumors and *n* = 20 analysis fields. Error bars indicate SD. Statistical significance was calculated using one-way ANOVA (****p < 0.0001). **D** Survival of 4NQO-L-tumor bearing WT mice treated with αPD-1, MRTX849, lapatinib, MRTX849/lapatinib, αPD-1/MRTX849, αPD-1/lapatinib, or a combination of αPD-1 and MRTX849/ lapatinib. **E** Top: Growth curves of 4NQO-L tumors treated with vehicle (*n* = 5 mice, 10 tumors), αPD-1 (*n* = 5 mice, 10 tumors), MRTX849 (*n* = 4 mice, 8 tumors), lapatinib (*n* = 5 mice, 10 tumors), MRTX849/lapatinib (*n* = 5 mice, 10 tumors), MRTX849/αPD-1 (*n* = 5 mice, 10 tumors), lapatinib/αPD-1 (*n* = 5 mice, 10 tumors), or a combination of MRTX849/lapatinib and αPD-1 (*n* = 5 mice, 10 tumors). Bottom: Fold changes in tumor volume after treatment with αPD-1, MRTX849, lapatinib, MRTX849/lapatinib, MRTX849/αPD-1, lapatinib/αPD-1, or the combination of MRTX849/ lapatinib and αPD-1

The observation that CD8^+^ T-cells influenced the efficacy of the MRTX849/lapatinib treatment without any evidence of tumor elimination motivated us to explore the involvement of programmed death ligand 1 (PD-L1) as a key negative regulator of CD8^+^ T-cell activity that facilitates disease progression to such combination therapy. Because PD-L1 expression on both tumor cells and cells in the TME can dampen T-cell activation and reinforce T-cell exhaustion [47–51] we initially performed IHC of PD-L1 staining in tumors after three days of treatment with MRTX849, lapatinib, or a combination of the two. IHC analysis of PD-L1 staining indicated a significant upregulation of PD-L1 following treatment with MRTX849 alone as well as in combination with lapatinib in both tumor cells and the tumor stroma (Fig. 5A). We confirmed the upregulation of PD-L1 in tumor cells (cytokeratin-14 positive) and non-tumor cells (cytokeratin-14 negative) by using immunofluorescence staining (Fig. 5B). Because cancer-associated fibroblasts (CAFs) are known to express PD-L1 [52, 53] and to suppress CD8^+^ T-cells [53] we stained the tumors with alpha-smooth muscle actin (αSMA), a CAF marker, and observed that both lapatinib and MRTX849 treatment induced massive accumulation of CAFs. However, similar to the accumulation of CD8^+^ T-cells, the accumulation of CAFs was greater in response to the combination of MRTX849/lapatinib vs. any of the monotherapies (Fig. 5C).

We therefore posited that treating mice with an immune checkpoint inhibitor targeting αPD-1 would attenuate immune suppression, ultimately leading to improved tumor clearance and prolonged response duration. To test this premise, we conducted an eight-arm experiment in which tumor-bearing mice were first treated with either IgG or αPD-1 (25 µg/kg), and then the different treatment groups received vehicle, MRTX849 (30 mg/kg), lapatinib (50 mg/kg), or a combination of the two agents. Mice treated with lapatinib, αPD-1, or lapatinib/αPD-1 combination regimens displayed tumor growth patterns similar to those of the vehicle-treated group (Fig. 5D and E). Consistent with our previous findings, MRTX849 monotherapy resulted in a temporary delay in tumor growth of approximately 10 days, whereas the duration of the response was doubled to ~ 20 days in response to the MRTX849/lapatinib combination. Supplementation of αPD-1 in combination with MRTX849 resulted in an improved response duration in 6 of 10 tumors (Fig. 5D and E) compared to MRTX849 monotherapy. However, eventually, only 2 of the 10 tumors demonstrated no tumor progression. Finally, treatment with MRTX849/lapatinib/αPD-1 resulted in dramatic tumor shrinkage in all tumors and led to long-term control in 8 of the 10 tumors. The degree of response at the endpoint of the experiment is shown in Fig. 5E. These results demonstrate that preventing PD-L1 suppression with αPD-1 further prolonged the duration of response and progression-free survival.

### MRTX849-acquired resistance tumors did not benefit from supplementation with αPD-1

**Fig. 6:**
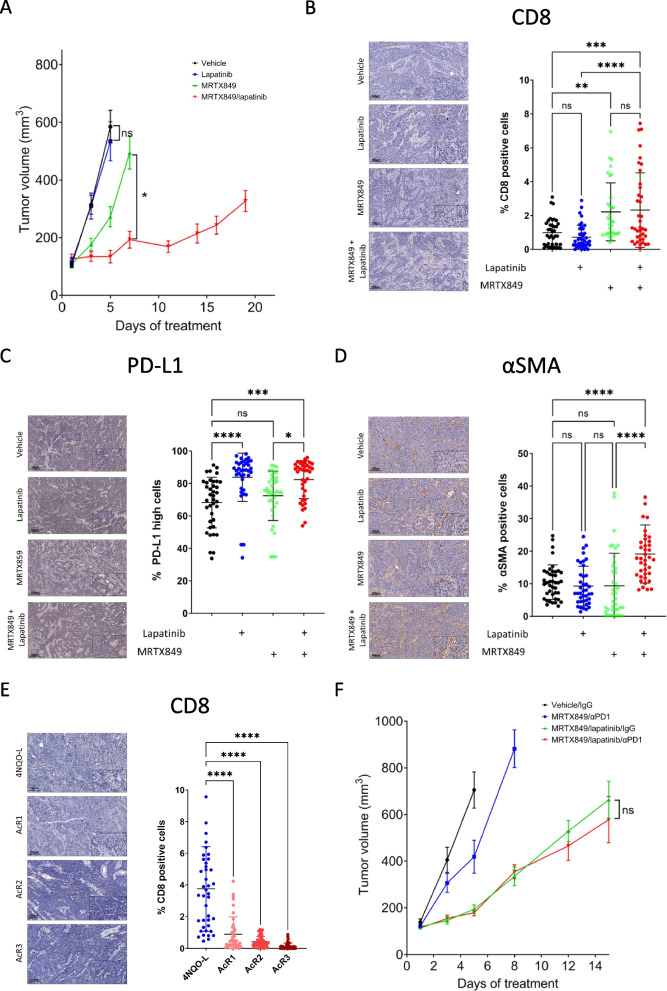
MRTX849/lapatinib/αPD-1 treatment is ineffective in MRTX849-resistant tumors. **A** Growth curve of 4NQO-L KRAS^G12C^i acquired resistant tumors in WT mice treated with vehicle, MRTX849, lapatinib or MRTX849/lapatinib combination (*n* = 6 mice, 12 tumors). **B** IHC images showing the infiltration of CD8^+^ T cells in the tissue sections of 4NQO-L KRAS^G12C^i acquired-resistance tumors treated with vehicle MRTX849, lapatinib, or MRTX849/lapatinib (scale bars: 100 µm; inset 10 µm). *n* = 4 tumors and *n* = 40 analysis fields. Error bars indicate SD. Statistical significance was calculated using one-way ANOVA (**p < 0.01, ***p < 0.001, ****p < 0.0001). **C** IHC images showing expression of PD-L1 in the tissue sections of 4NQO-L KRAS^G12C^i acquired resistant tumors treated with vehicle, MRTX849, lapatinib or MRTX849/lapatinib (scale bars: 100 µm; inset 10 µm). *n* = 4 tumors and *n* = 38 analysis fields. Error bars indicate SD. Statistical significance was calculated using one-way ANOVA (*p < 0.05, ***p < 0.001, ****p < 0.0001), ns denotes not significant. **D** IHC images showing the expression of αSMA in the tissue sections of 4NQO-L KRAS^G12C^i acquired resistant tumors treated with vehicle, MRTX849, lapatinib or MRTX849/lapatinib (scale bars: 100 µm; inset 10 µm). *n* = 4 tumors and *n* = 40 analysis fields. Error bars indicate SD. Statistical significance was calculated using one-way ANOVA (****p < 0.0001), ns denotes not significant. **E** IHC images showing the infiltration of CD8^+^ T-cells in the tissue sections of 4NQO-L KRAS^G12C^i-acquired-resistance tumors (scale bars: 100 µm; inset 10 µm). *n* = 4 tumors and *n* = 41 analysis fields. Error bars indicate SD. Statistical significance was calculated using one-way ANOVA (****p < 0.0001). **F** Growth curves of 4NQO-L KRAS^G12C^i-acquired-resistance tumors treated with IgG, IgG/MRTX849/lapatinib, MRTX849/αPD-1, or the combination of MRTX849/ lapatinib and αPD-1 (*n* = 6 mice, 12 tumors). Statistical significance was calculated using one-way ANOVA, ns denotes not significant

To uncover if intrinsic resistance to KRAS^G12C^i influences the composition of the TME and thus the response to therapy, we explored the response of KRAS^G12C^i-acquired-resistance 4NQO-L-AcR1 cells to the MRTX849/lapatinib combination in vivo and investigated whether CD8^+^ T cells were involved in the efficacy of this model. To evaluate the antitumor activity of MRTX849, lapatinib, and their combination in vivo, 4NQO-L-AcR1 cells were implanted s.c. into the flanks of C57BL/6 immune-competent mice. Tumor-bearing mice were randomized into four groups and treated daily with vehicle, MRTX849 (30 mg/kg/day), lapatinib (50 mg/kg/day), or a combination of the two agents. Mice treated with MRTX849 or lapatinib showed tumor progression, while the combined treatment delayed tumor growth, with a durable response for 15 days (Fig. 6A). IHC staining for CD8 revealed that the combined treatment significantly enhanced the infiltration of CD8^+^ T-cells into the TME (Fig. 6B). However, despite the increase in CD8^+^ T- cells in the tumor, their percentage in the tumor remained low, reaching an average of 2% in the mice treated with MRTX849 and or the combination of MRTX849/lapatinib. Further profiling of the tumors for PD-L1 expression (Fig. 6C) showed a high level of PD-L1 in all tumors (including before treatment), with ~ 70% of the cells staining positive and the percentage increasing to 80% following treatment with MRTX849/lapatinib. A modest increase in the accumulation of CAFs was detected only in the combination group compared to that in the vehicle-treated group (Fig. 6D).

To explore whether a low number of CD8^+^ T cells was associated with the acquisition of resistance KRAS^G12C^i, we injected each of the three KRAS^G12C^i-acquired-resistance cell line models into WT mice, and then stained the tumors that developed for CD8. IHC analysis confirmed that acquired-resistance tumors exhibited less infiltration of CD8^+^ T-cells (Fig. 6E), which may indicate that the acquisition of resistance to KRAS^G12C^i is associated with the ability to induce immune suppression. The low number of CD8^+^ T-cells in KRAS^G12C^i-acquired-resistance tumors, and after treatment with MRTX849/lapatinib (Fig. 6B) led to the hypothesis that the contribution of CD8^+^ T-cells to the efficacy of MRTX849/lapatinib may be limited. To test that, we enhanced CD8^+^ T-cell activity using αPD-1 in mice treated with the MRTX849/lapatinib combination and followed tumor growth. Specifically, 4NQO-L-AcR1 tumor-bearing mice were treated with a combination of lapatinib (30 mg/kg) and MRTX849 (50 mg/kg), concomitantly with either IgG or αPD-1. Tumor growth kinetics showed that supplementation of MRTX849/lapatinib with αPD-1 did not improve the efficacy of the MRTX849/lapatinib combination (Fig. 6F).

**Fig. 7:**
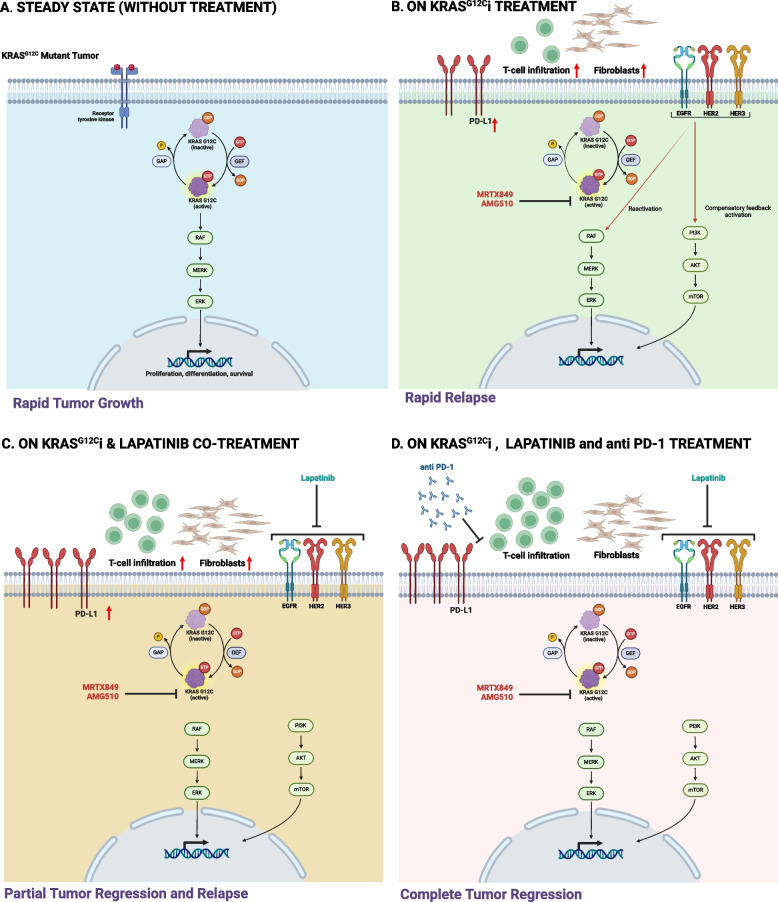
Schematic representation of the intrinsic and extrinsic response of 4NQO-L cells during MRTX849, MRTX849/lapatinib, and MRTX849/lapatinib/αPD-1 treatment. **A** Steady state (without treatment): KRAS^G12C^ constitutive activation leads to MAPK and survival cellular signaling, causing rapid tumor proliferation. **B** On KRAS^G12C^i treatment: During treatment with the KRAS^G12C^i, MRTX849 or AMG510, the activation of KRAS^G12C^ is inhibited, but upregulation of pan-HERs and PD-L1 occurs, along with fibroblast accumulation. The upregulation and activation of pan-HERs lead to activation of the PI3K-AKT-mTOR pathway and reactivation of the MAPK pathway. This feedback activation leads to rapid tumor relapse. **C** On KRAS^G12C^i and lapatinib co-treatment: Both KRAS^G12C^ activation and HER-mediated PI3K-AKT-mTOR and MAPK pathway activation are inhibited, leading to reduced tumor proliferation but sustained PD-L1 upregulation and accumulation of fibroblasts. **D** On KRAS^G12C^i, lapatinib, and αPD-1 treatment: Both KRAS.^G12C^ activation and the PI3K pathway are inhibited, and the use of αPD-1 prevented PD-L1 mediate immune suppressive activity, making the tumor cells vulnerable to treatment and hence causing complete regression. (This illustration is created using Biorender.com)

Taken together, our findings indicate that treatment with KRAS^G12C^i results in increased expression of the intrinsic mechanism of resistance pan-HERs and immune escape mechanisms including PD-L1 by tumor cells, and concomitant accumulation of CAFs that presumably suppress the activity of the infiltrated CD8^+^ T cells. The upregulation and activation of pan-HER result in the activation of the PI3K-AKT-mTOR pathway and reactivation of the MAPK pathway and consequently rapid tumor progression and treatment resistance. Blocking the pan-HERs with lapatinib in combination with MRTX849 leads to partial regression of the tumors, but tumor relapse occurs due to an immune suppressive environment that limits CD8^+^ T-cell activity. Remarkably, supplementation of the MRTX849/lapatinib combination with αPD-1 induces complete and durable regression of the KRAS^G12C^i-sensitive tumors (Fig. 7). However, supplementation of αPD-1 to MRTX849/lapatinib had no benefit in KRAS^G12C^i -resistant tumors which may related to the immune-desert phenotype of these tumors.

## Discussion

Genomic profiling of tumors has facilitated the therapeutic application of targeted therapies in HNC [54]. However, targeted therapies have limited efficacy in unselected HNC cases, and therefore the European Organization for Research and Treatment of Cancer (EORTC) initiated a biomarker-driven study of HNC, employing molecular profiling to guide personalized treatments [55]. As observed approximately 20% of HNC cases exhibit mutations in the MAPK pathway, particularly in RAS family members [56]. *KRAS* is the second most commonly altered member of the RAS family in patients with HNC, with a mutational rate ranging from 1.7% to 12.7% [57–61]. Additionally, germline mutations occur in the 3' untranslated region (3' UTR) of *KRAS* at an incidence of 15% to 32% in HNC patients [58, 59]. Both types of *KRAS* abnormalities can result in poor prognosis and resistance to cetuximab [58–60] These data suggest that individuals with KRAS mutation are eligible for basket trials with specific KRAS inhibitors, specifically small molecules that block KRAS with G12D or G12C mutations [62–65] Indeed, basket clinical trials (including NCT03785249, NCT04380753, NCT04449874, NCT05737706, and NCT05382559) have therefore targeted KRAS mutations using promising inhibitors also for HNC patients, such as sotorasib and adagrasib. Currently, the progression-free survival with sotorasib was only 6.3 months, and only 45% of patients showed a partial response to adagrasib [17]. Although resistance to these inhibitors has already emerged as a serious concern [18, 21, 22, 24, 66–72], it is essential to have a comprehensive understanding of the underlying resistance mechanisms (both intrinsic and extrinsic).

In this study, we took advantage of the only known KRAS^G12C^ mutant murine HNC cell line, 4NQO-L, to study the response and resistance to KRAS^G12C^i in an immunocompetent murine system. To this end, we investigated the tumor cell autonomous (intrinsic) response and the response of the TME (extrinsic), both of these responses are critical for therapeutic efficacy in HNC [16]. Although it has previously been shown that there is a dose-dependent inhibition of MAPK activation by MRTX849 [30], our study demonstrated that MRTX849 (or AMG510) caused time-dependent activation of a compensatory survival machinery through the upregulated and enhanced phosphorylation of HER family members. This adaptive intrinsic resistance mechanism, which has been widely observed with treatments with KRAS^G12C^i in various other cancer types [17, 23, 33, 34, 73], is consistent with the resistance pattern observed for MEK inhibition in HNC [74]. It is known that decreased RAS signaling results in the loss of feedback and an increase in upstream signaling pathways through the activation of growth factor receptors [26, 75] Activation of RAS pathway can trigger signaling through wild-type HRAS or NRAS proteins expressed in all cancer cells with mutant KRAS or through wild-type KRAS in cancer cells that retain the wild-type KRAS allele alongside the oncogenic mutant [75] Our study also revealed that the overexpression of EGFR/HER2 limited the efficacy of KRAS^G12C^i—a finding that is consistent with the clinical outcome in a sotorasib-treated patient with KRAS^G12C^ lung cancer where clinical resistance to KRAS^G12C^ inhibition is linked to acquired HER2 upregulation [33] and also the in vitro and clinical finding that EGFR blockade reverses resistance to KRAS^G12C^ inhibition in colorectal cancer [34, 73, 76–79] Our result also revealed that overexpressing HER3 alone does not impart resistance to KRAS^G12C^i but studies have shown that HER3 overexpression is often associated with overexpression of HER2 or EGFR, playing an important role as co-receptor in HER2^+^ cancers – implicating in resistance to therapies targeting other HER receptors as well as in resistance to chemotherapies [80].

Our findings that supplementation with EGF limited the efficacy of MRTX849 emphasize the prominent role of HER1/2 in imparting KRAS^G12C^i resistance. This observation is consistent with that of Xue et al. [24], who demonstrated that stimulation of ARS 1620-treated cells with EGF resulted in reactivation of KRAS, strongly suggesting that EGFR mediates adaptive resistance to KRAS^G12C^i. HERs may influence the adaptation to treatment with KRAS^G12C^i through two different mechanisms: (i) promoting SOS1/2-mediated nucleotide exchange to activate RTK and shift KRAS^G12C^ to its GTP-bound form, which is no longer sensitive to the drug, or (ii) circumventing inhibition in a G12C-independent manner, for example, through the activation of wild-type RAS, PI3K/AKT/mTOR, or other pathways [81–83]. In light of these ideas, we analyzed the additive effects of various RTK inhibitors on MRTX849 treatment. Importantly, we found that a strategy targeting a single RTK to block adaptive resistance may be ineffective. However, we observed that co-targeting of EGFR and HER2 abrogated feedback reactivation of AKT signaling following KRAS^G12C^ inhibition and that a combination of a KRAS^G12C^ inhibitor (MRTX849 or AMG510) and the pan-HER inhibitor lapatinib led to sustained RAS pathway suppression and improved efficacy in vitro and in vivo (Figs. 2F and 4A).

Our data generated from experiments with acquired-resistance cells also indicated that pan-HER overexpression and persistent activation of the MAPK pathway are critical survival mechanisms adopted during the acquisition of resistance to KRAS^G12C^i by KRAS^G12C^ mutated cells. This observation is in line with previous findings of rapid feedback reactivation of RAS pathway signaling in KRAS^G12C^ cancer models following treatment with KRAS^G12C^i [23, 28, 83] In our model, HER-driven feedback may contribute to RAS reactivation through increased cycling of KRAS^G12C^ to its active GTP-bound form or by the induction of wild-type RAS in a KRAS^G12C^-independent manner. Another notable finding of our study was that MRTX849-resistant cells were also resistant to AMG510 and vice versa. The cross-resistance between MRTX849 and AMG510 suggests that these resistant clones follow a common mechanism during the process of acquiring resistance, even though the two compounds are structurally different. The study of Koga et al. demonstrated that secondary Y96D and Y96S mutations in KRAS caused cross-resistance to both sotorasib and adagrasib [68] Analysis of the secondary mutations in our resistant clones is warranted for better insights into the genetic mechanism of acquired resistance to KRAS^G12C^ inhibition.

The studies of Patricelli et al. [84], Lito et al. [85] and Ryan et al. [83] demonstrated that pretreatment of cells with either erlotinib or afatinib, or concurrent inhibition of c-MET, SRC, or FGFR, can prime cells for KRAS inhibition by KRAS^G12C^i. Additionally, the phospho-RTK array analysis of Ryan et al. [83] revealed that multiple RTKs are involved in RAS reactivation, and high-throughput drug screening by Misale et al. [81] identified several RTK inhibitors that exhibit strong synergies with ARS-1620, a KRAS^G12C^i. However, these synergistic effects were inconsistent across different cell models, suggesting that strategies targeting a single RTK in combination with KRAS^G12C^ inhibition may not be universally effective for cancer therapy. In our KRAS^G12C^ mutant model, targeting pan-HER in combination with KRAS^G12C^i was found to represent an attractive strategy to suppress the adaptive mechanisms of resistance and to delay tumor growth in vivo.

Studies conducted in genetically engineered murine models exhibiting spontaneous tumors driven by mutated KRAS have demonstrated that oncogenic KRAS orchestrates an immunosuppressive TME [86, 87] In these models, KRAS activation leads to increased secretion of IL23, CCL9, VEGFA, and CXCL3 by tumor cells, which recruit immunosuppressive macrophages and myeloid-derived suppressor cells (MDSCs) into the TME, resulting in the exclusion of adaptive T and B cells in a PD-L1–dependent manner. Initial reports of treatment with KRAS^G12C^i showed that durable responses in mice are T-cell dependent [45, 46] These studies have shown that KRAS^G12C^i stimulates antitumor immunity by inducing a proinflammatory microenvironment enriched with tumor-suppressive M1 macrophages and cytotoxic CD8^+^ T cells via the production of CXCL10/11 [45, 46] Mechanistically, it is now known that KRAS^G12C^i treatment up-regulates interferon signaling via Myc inhibition, leading to reduced tumor infiltration by immunosuppressive cells, enhanced infiltration and activation of cytotoxic T-cells, and increased antigen presentation [44]. In our model, we observed infiltration of CD8^+^ T-cells upon MRTX849 treatment, combination therapy with lapatinib further improved or enhanced the infiltration of CD8^+^ T-cells, providing a durable response to treatment. In animal breast cancer models lapatinib promotes tumor infiltration by CD4^+ ^CD8^+^ IFN-γ-producing T-cells through a Stat1-dependent pathway, suggesting that this immune activation can play a role in lapatinib antitumor activity [88] Fedele et al. [89] showed that a combination of SHP2 and KRAS^G12C^i awarded good tumor control and increased T cell infiltration in an orthotopic model of lung cancer. The enhanced infiltration of CD8 T cells in MRTX849/lapatinib-treated mice, along with CD8^+^ T cell depletion studies, showed augmented antitumor activity in CD8-intact mice, providing strong evidence that adaptive antitumor immune activity is required for a prolonged response.

In MRTX849-responsive tumors, we found that MRTX849 alone or MRTX849/lapatinib treatment increased the expression of PD-L1 in both the tumor and the surrounding tissue. This observation led us to evaluate the efficacy of αPD-1 therapy in combination with MRTX849/lapatinib. The results for MRTX849, αPD-1, and their combination in the KRAS^G12C^ 4NQO-L model were consistent with those of previous studies that investigated the pharmacodynamic and antitumor effects of the KRAS^G12C^i, AMG510 [45] and MRTX849 [46] either alone or in combination with αPD-1 therapy. Although MRTX849/lapatinib led to significant regression in immune-competent tumor models, durable complete tumor regression responses were observed only by adding αPD-1 to the MRTX849/lapatinib combination therapy, suggesting that further enhancement of the antitumor immune response by blocking the PD-1 pathway may be critical for providing long-term disease control in patients. Currently, many clinical trials are underway to assess the combinations of αPD-1 pathway immune checkpoint blockers and different KRAS.^G12C^i comprising RTK inhibitors and agents targeting pan-KRAS (for example, SOS1 and SHP2 inhibitors) [44, 89, 90]

We found that targeting HER effectively suppressed resistance in our KRAS^G12C^ mutant murine HNC KRAS^G12C^i-sensitive cell line. Therefore, we hypothesized that targeting HER-mediated feedback reactivation during acquired resistance would break the critical feedback loop at its earliest point and render resistant cells sensitive to KRAS^G12C^ inhibition. Indeed, we found that targeting HER with lapatinib in combination with MRTX849 improved treatment efficacy for MRTX849-resistant tumors. MRTX849-resistant tumors showed high baseline expression of PD-L1 and hence did not respond to MRTX849/lapatinib/αPD-1 treatment (Figure S4). Numerous investigations have demonstrated that the simultaneous use of KRAS^G12C^ suppressors and PD-L1 therapy is only effective in highly immunogenic tumors, with many of these tumors eventually becoming resistant to the treatment [44, 91, 92]. Recently, emerging evidence has suggested that activation of the RTK signaling pathway can induce PD-L1 expression in HNC [93, 94] Thus, combination strategies of KRAS^G12C^i with pan-HER inhibitors may be efficacious in preventing the onset of acquired drug resistance and improving CD8^+^ T-cell infiltration, thus providing a therapeutic opportunity for immunotherapy. Another important finding in our study is the dynamic and diverse patterns of remodeling within the stroma, such as the accumulation of CAFs, reduced infiltration of immune cells, and upregulation of PD-L1 expression. Contrary to the findings in the sensitive model, MRTX849-resistant tumors were transformed into immunologically cold entities and showed a significant decline in adaptive immune cell populations. These results align with those of a previous syngeneic CT26 colon cancer model, which demonstrated that AMG510-resistant tumors were characterized by immune escape and reduced numbers of adaptive immune cells, leading to an immunosuppressive tumor microenvironment [22]. Thus, future studies should investigate the potential role of immune escape in MRTX849 resistance.

As indicated above, CAFs are important players in the TME. These cells make a crucial contribution to cancer-related inflammation by interacting with cancer cells and other immune cells in the TME, thus promoting cancer cell growth, survival, angiogenesis, and suppressing anti-tumor immune responses. Various factors, including hypoxia, chemokines, cytokines, and metabolic products of cancer cells, are involved in activating these cells and determining their functional polarization [95]. The accumulation of CAFs as a means of resistance to targeted therapies has been well-documented in several studies that have focused on the inhibition of the RAS/MAPK/MEK pathways [11, 29]. Furthermore, recent research suggests that these fibroblast cells also have a significant impact on tumor metabolism [96] and may be responsible for the transition from a "hot" to a "cold" tumor immune phenotype [97] with the transition constituting a major challenge in cancer immunotherapy. A recent study demonstrated that blocking the oncogenic KRAS gene led to an increase in the expression of HER2 and HER3 in both human and mouse pancreatic cancer models, which led cancer cells to rely on NRG1 secreted by CAFs as a critical factor for survival [98] such a mechanism involving CAFs might also functional during KRAS^G12C^ inhibition in our model, which determines treatment response.

## Conclusions

Overall, our study demonstrated that pan-HER inhibition along with the activity of MRTX849 could curtail adaptive resistance, and the use of αPD-1 therapy together with this combination could prolong progression-free survival in KRAS^G12C^i-responsive tumors. Our results highlight the importance of supplementation of αPD-1 therapy concurrently with MRTX849/lapatinib is critical for durable and prolonged response rather than performing sequential treatment after resistance is established. Finally, our findings emphasize the significance of exploring the diverse aspects of tumor biology, particularly the immune system, regulated by mutant KRAS to create logical combinations that could produce durable patient responses.

## Limitations

Although our study provides new insights into the intrinsic and extrinsic mechanism of resistance development to KRAS^G12C^i, it also has a few shortcomings. The use of a single KRAS^G12C^ murine HNC model and the lack of studies on other immune cells, such as MDSCs, tumor-associated macrophages, natural killer cells, and B cells, during tumor response, relapse, and resistance all constitute limitations. Furthermore, the genomic sequencing of resistant lines could provide an alternative genetic mechanism for resistance. These shortcomings will be addressed in our ongoing research directed at a better understanding of how KRAS^G12C^i modulates the stromal microenvironment during adaptive and acquired resistance.

## Supplementary Information

Supplementary Material 1.

Supplementary Material 2.

Supplementary Material 3.

## Data Availability

All data generated or analysed during this study are included in this published article [and its supplementary information files].

## Abbreviations

αSMA: Alpha Smooth Muscle Actin
CAF: Cancer Associated Fibroblast
CXCL: Chemokine (C-X-C motif) Ligand
EGF: Epidermal Growth Factor
EGFR: Epidermal Growth Factor Receptor
GTP: Guanosine Triphosphate
HRAS: Harvey Rat Sarcoma Viral Oncogene Homolog
HNC: Head and Neck Cancer
HER: Human Epidermal Growth Factor Receptor
IFNγ: Interferon gamma
IgG: Immunoglobulin G
IHC: Immunohistochemistry
KRAS: Kirsten Rat Sarcoma Viral Oncogene Homolog
MDSC: Myeloid Derived Suppressor Cell
NRG: Neuregulin-beta 1;
PD-1: Programmed Cell Death Protein -1
PD-L1: Programmed Death-Ligand 1
RTK: Receptor Tyrosine Kinase
SHP1: Src Homology region 2 domain-containing Phosphatase-1
SOS1: Son of Sevenless 1
TME: Tumor Microenvironment
VEGF: Vascular Endothelial Growth Factor
