# Supplementary material for: Dual inhibition of HERs and PD-1 counteract resistance in KRASG12C-mutant head and neck cancer
Source: J Exp Clin Cancer Res. 2024 Nov 20;43:308. doi: 10.1186/s13046-024-03227-0 (PMC11577641; doi:10.1186/s13046-024-03227-0)
Supplement: Supplementary file 1 — Supplementary Material 1. [file 13046_2024_3227_MOESM1_ESM.docx]

| **Supplementary Table 1: IC_50_ values of various treatments** | | |  |  |  |  |
| --- | --- | --- | --- | --- | --- | --- |
| **Figure** | **Cell line** | **Drug** | **IC_50_ value** | **Std Error (LogIC_50_)** |  |  |
| 1A | 4NQO-L | MRTX849 | 0.03374 | 0.1416 |  |  |
| 1A | 4NQO-T | MRTX849 | 1.025 | 0.1021 |  |  |
| 1A | 4NQO-L | AMG510 | 0.4662 | 0.07642 |  |  |
| 1A | 4NQO-T | AMG510 | 43.57 | 0.051 |  |  |
| 1D | 4NQO-L, Cont for EGFR | MRTX849 | 0.9716 | 0.1276 |  |  |
| 1D | 4NQO-L, EGFR | MRTX849 | 3.343 | 0.07115 |  |  |
| 1D | 4NQO-L, Cont for HER2 | MRTX849 | 0.001831 | 0.1402 |  |  |
| 1D | 4NQO-L, HER2 | MRTX849 | 1.777 | 0.1117 |  |  |
| 1D | 4NQO-L, Cont for HER3 | MRTX849 | 0.001365 | 0.1273 |  |  |
| 1D | 4NQO-L, HER3 | MRTX849 | 0.0007921 | 0.1578 |  |  |
| 1E | L121 | MRTX849 | 0.4498 | 0.07916 |  |  |
| 1E | L206 | MRTX849 | 1.024 | 0.0576 |  |  |
| 1E | L4 | MRTX849 | 0.002734 | 0.1532 |  |  |
| 1E | L110 | MRTX849 | 0.002866 | 0.09288 |  |  |
| 1E | L15 | MRTX849 | 1.179 | 0.09104 |  |  |
| 1E | L11 | MRTX849 | 0.1051 | 0.134 |  |  |
| 1E | L9 | MRTX849 | 0.006694 | 0.1492 |  |  |
| 1E | L202 | MRTX849 | 0.1832 | 0.06272 |  |  |
| 1E | L205 | MRTX849 | 0.01429 | 0.1285 |  |  |
| 1E | L113 | MRTX849 | 0.02124 | 0.08101 |  |  |
| 1E | L30 | MRTX849 | 0.0294 | 0.09872 |  |  |
| 1E | L207 | MRTX849 | 0.1723 | 0.07659 |  |  |
| 2C | 4NQO-L | LAPATINIB | 0.6626 | 0.06729 |  |  |
| 2C | 4NQO-L | LAPATINIB + MRTX849 | 0.02181 | 0.1358 |  |  |
| 2C | 4NQO-L | ERLOTINIB | 0.8775 | 0.06107 |  |  |
| 2C | 4NQO-L | ERLOTINIB + MRTX849 | 0.2713 | 0.109 |  |  |
| 2C | 4NQO-L | Afatinib | 0.1092 | 0.08453 |  |  |
| 2C | 4NQO-L | Afatinib + MRTX849 | 0.00445 | 0.1468 |  |  |
| 2C | 4NQO-L | PHA | 3.397 | 0.1444 |  |  |
| 2C | 4NQO-L | PHA + MRTX849 | 2.038 | 0.07508 |  |  |
| 2C | 4NQO-L | Foretinib | 0.8847 | 0.07398 |  |  |
| 2C | 4NQO-L | Foretinib + MRTX849 | 0.5881 | 0.09502 |  |  |
| 2C | 4NQO-L | R428 | 5.344 | 0.1444 |  |  |
| 2C | 4NQO-L | R428 + MRTX849 | 7.394 | 0.2009 |  |  |
| 3A | 4NQO-L | MRTX849 | 0.006289 | 0.1597 |  |  |
| 3A | 4NQO-L-AcR1 | MRTX849 | 0.5225 | 0.08854 |  |  |
| 3A | 4NQO-L-AcR2 | MRTX849 | 0.4755 | 0.05916 |  |  |
| 3A | 4NQO-L-AcR3 | MRTX849 | 0.2504 | 0.06215 |  |  |
| 3A | 4NQO-L | AMG510 | 0.1326 | 0.1036 |  |  |
| 3A | 4NQO-L-AcR1 | AMG510 | 16.08 | 0.1205 |  |  |
| 3A | 4NQO-L-AcR2 | AMG510 | 10.69 | 0.1036 |  |  |
| 3A | 4NQO-L-AcR3 | AMG510 | 3.32 | 0.07726 |  |  |
