# Supplementary figures and images for: Dual inhibition of HERs and PD-1 counteract resistance in KRASG12C-mutant head and neck cancer
Source: J Exp Clin Cancer Res. 2024 Nov 20;43:308. doi: 10.1186/s13046-024-03227-0 (PMC11577641; doi:10.1186/s13046-024-03227-0)

## Slide 1
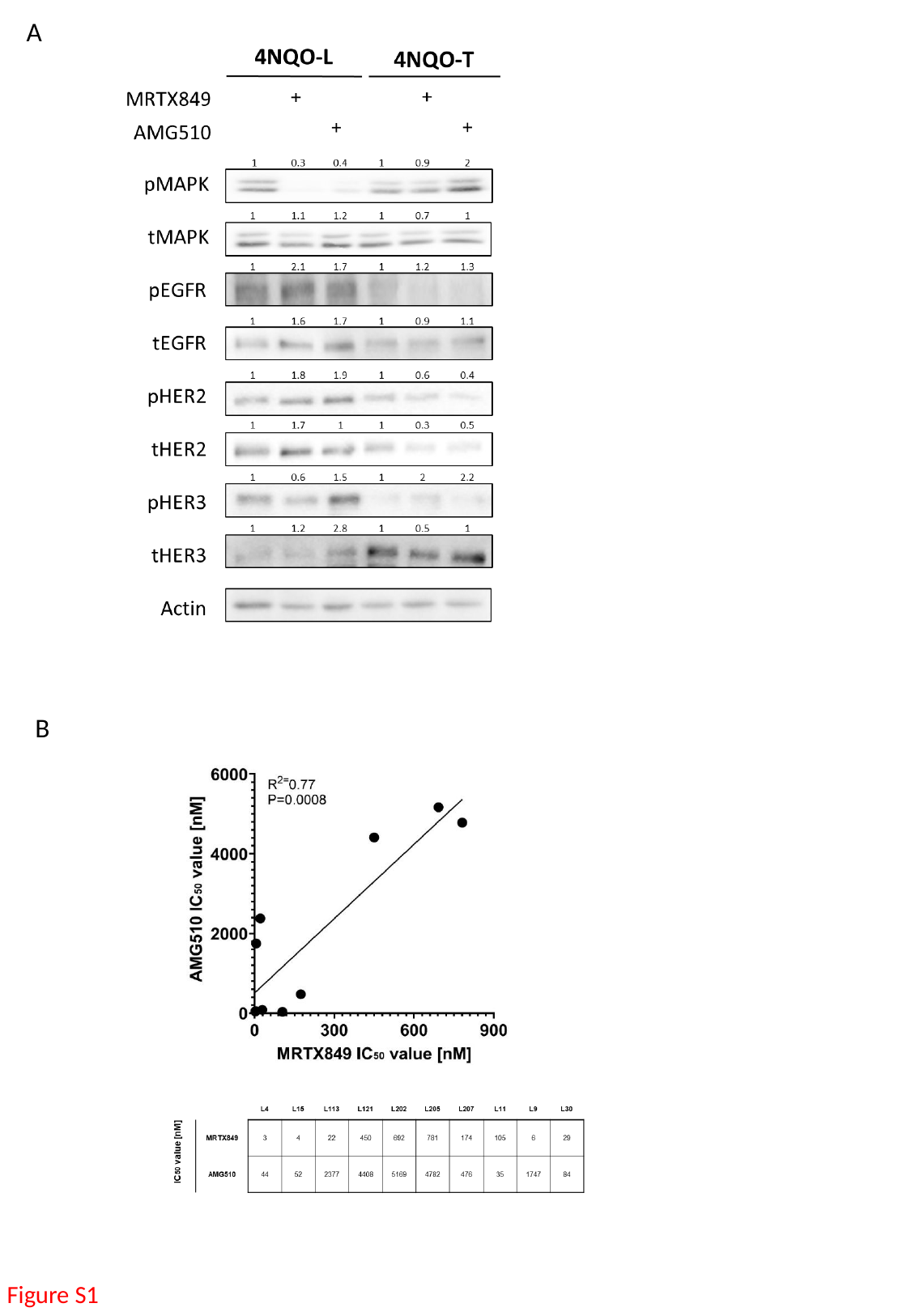

A
B
Figure S1

## Slide 2
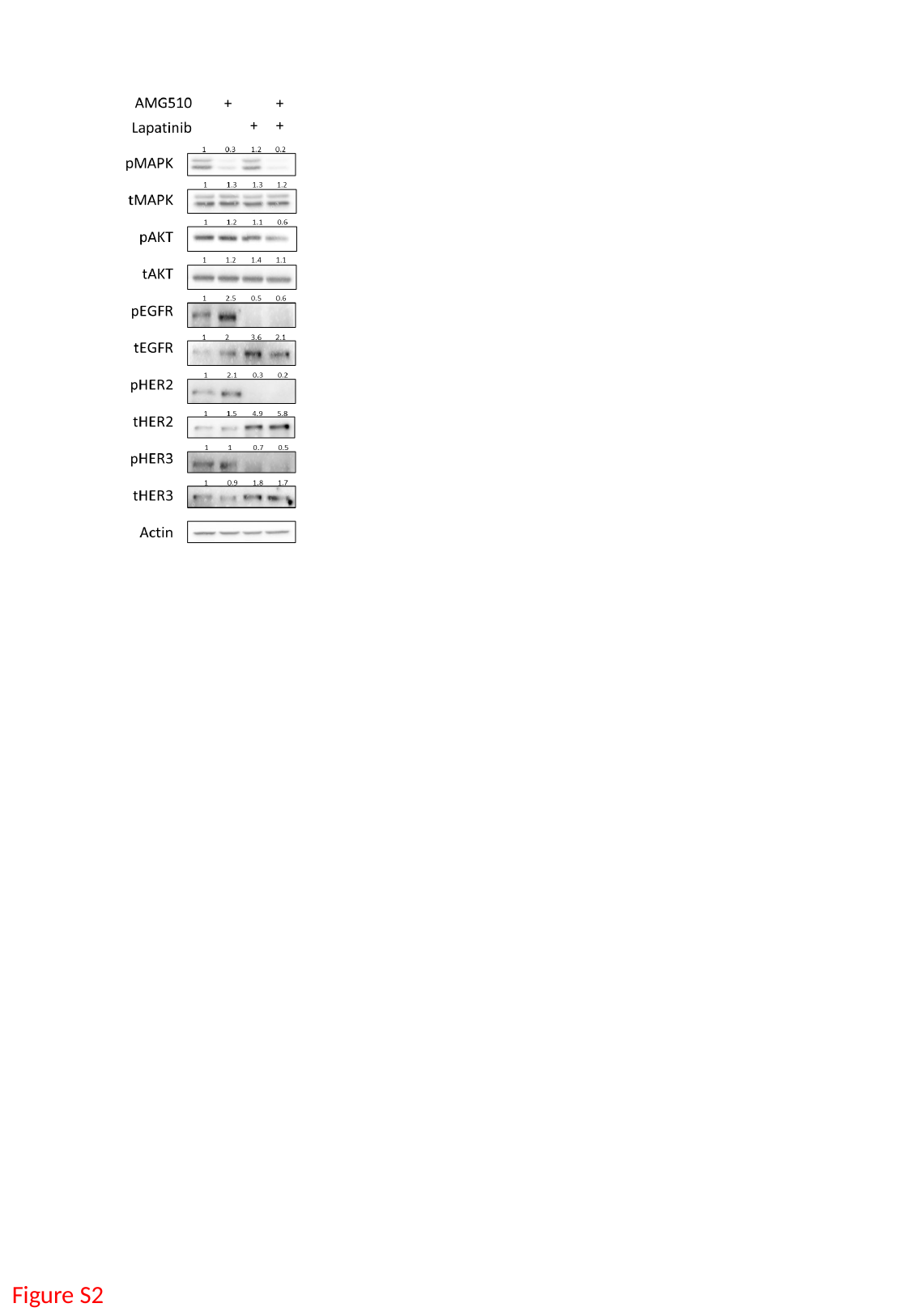

Figure S2

## Slide 3
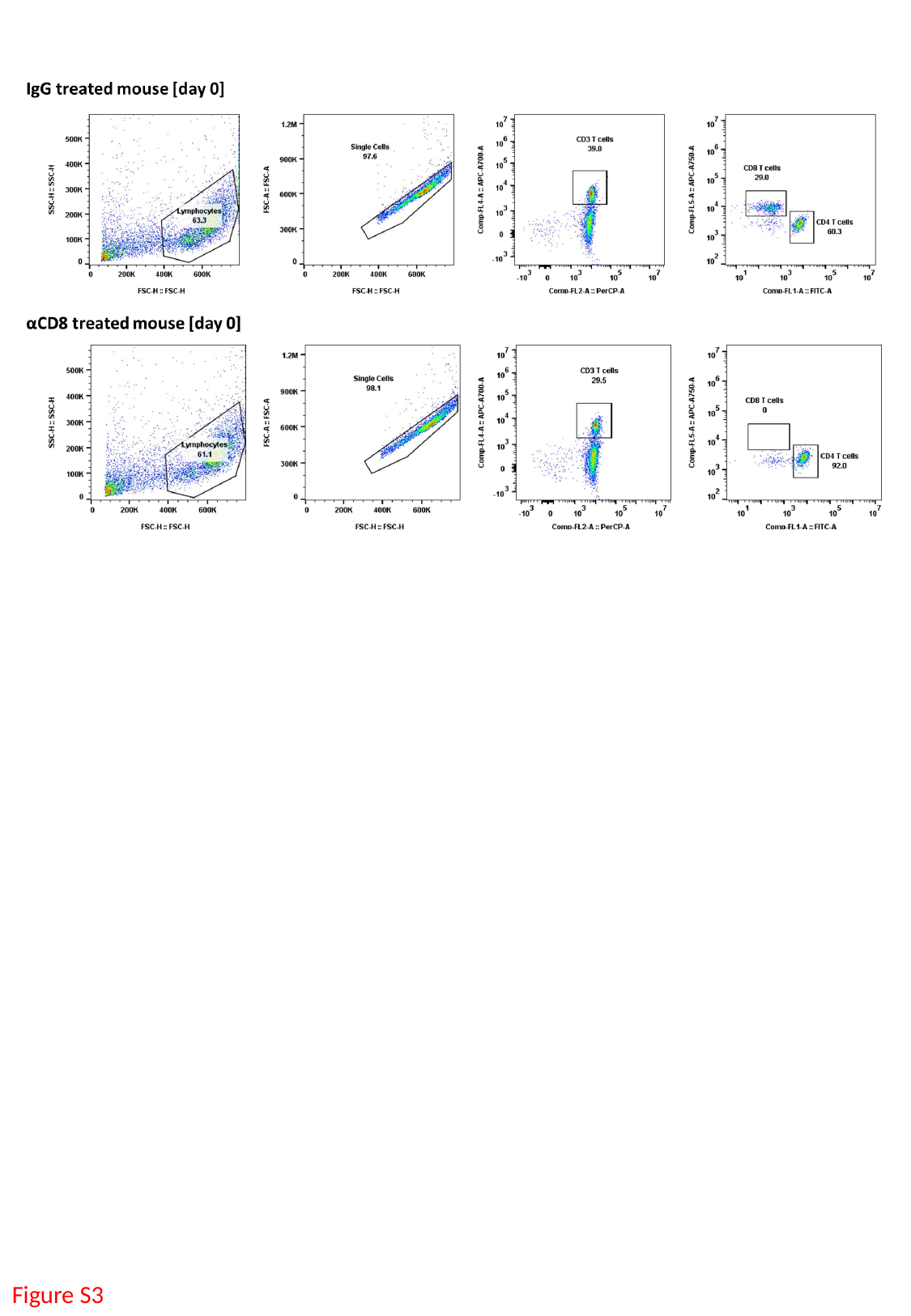

Figure S3

## Slide 4
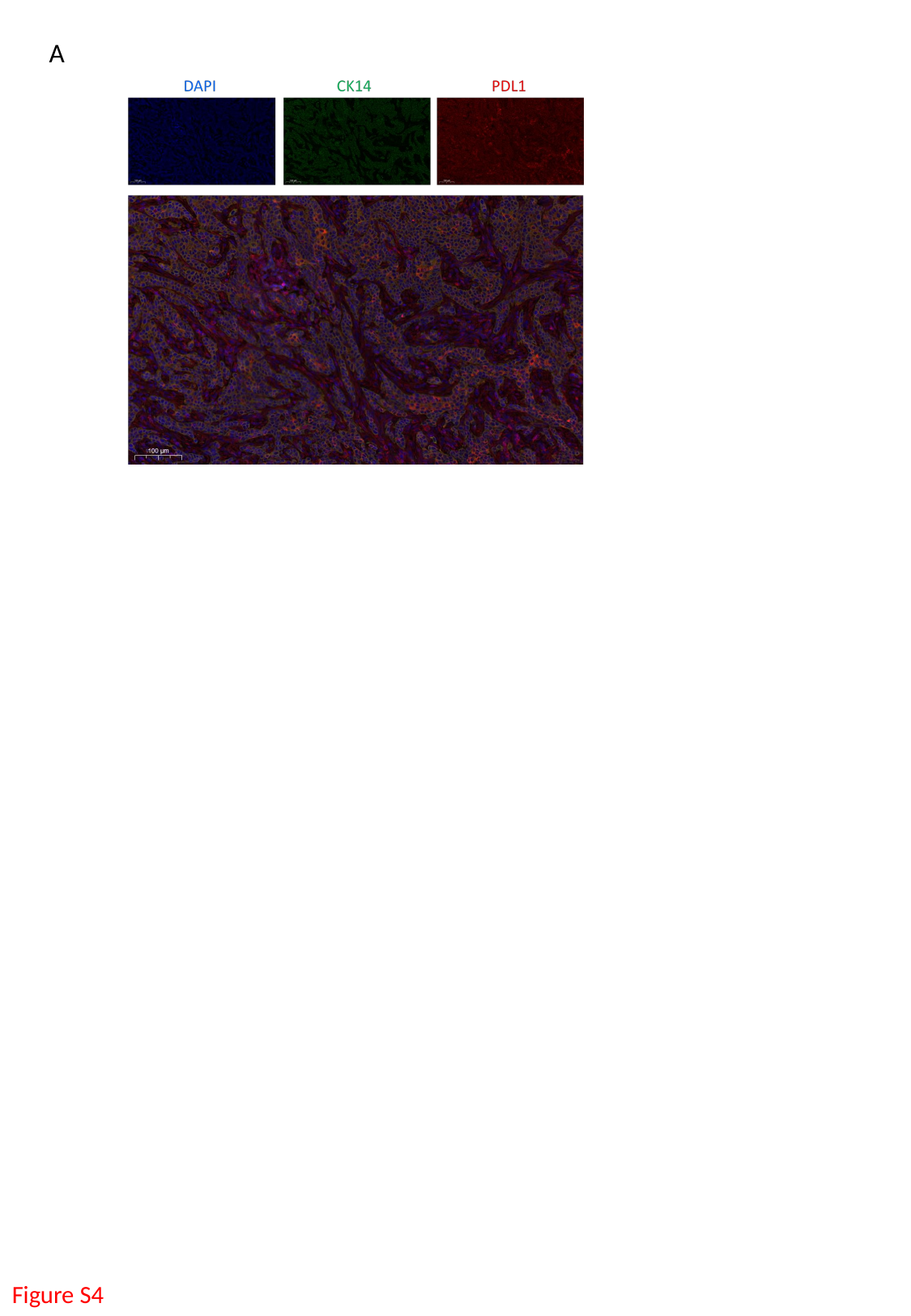

A
Figure S4

Supplement: Supplementary file 2 — Supplementary Material 2. [file 13046_2024_3227_MOESM2_ESM.pptx]
